# Daily, seasonal, and long-distance movements inferred from Fastloc-GPS telemetry of immature green turtles *(Chelonia mydas)* at a high-latitude, mid-ocean developmental site

**DOI:** 10.1371/journal.pone.0292235

**Published:** 2023-12-15

**Authors:** Robert F. Hardy, Anne B. Meylan, Jennifer A. Gray, Peter A. Meylan

**Affiliations:** 1 Fish and Wildlife Research Institute, Florida Fish and Wildlife Conservation Commission, St. Petersburg, Florida, United States of America; 2 Bermuda Zoological Society, Flatts, Bermuda; 3 Natural Sciences, Eckerd College, Saint Petersburg, Florida, United States of America; Deakin University, AUSTRALIA

## Abstract

To characterize the movements and habitat use of juvenile green turtles (*Chelonia mydas*) in benthic developmental habitat, we deployed Fastloc-GPS-enabled satellite transmitters on 16 individuals captured as part of a multi-decade study of green turtles on the Bermuda Platform. We characterized residence areas, distinct use areas within them, and seasonal movements based on an average of 562 Fastloc-GPS positions and 284 tracking days per turtle. We estimated residence area sizes using traditional home range methods, *e*.*g*., 90% utilization distribution (UD) (mean 2.29 ±2.71 km^2^) and 50% UD (mean 0.54 ±0.69 km^2^). Total residence area size increased significantly over the 8-year study, from <1 km^2^ before 2013 to ≥3 km^2^ in 2018 (R^2^ = 0.51, F1,14 = 14.55, p = 0.0019), corresponding to a period of decline in seagrass habitat and suggesting increased foraging effort. We identified three types of distinct use areas within residence areas where tracked turtles typically exhibited behavioral fidelity: foraging, resting, and cool weather refugia. These distinct use areas were smaller than high-use areas from previous studies; *e*.*g*., seagrass meadow foraging areas averaged 0.05 km^2^. Most turtles made daily transits between foraging and resting sites; for some individuals, these involved crossing frequently used vessel navigation channels. Seasonal variation in behavior suggested that the overwintering strategy for green turtles on the Bermuda Platform involves “optional dormancy,” during which turtles spent less time on seagrass meadows and made brief excursions to distinct deeper habitats. Four individuals made directed (mean path straightness = 0.93 ±0.02 SD) developmental migrations away from Bermuda toward known adult foraging range. Results of our study further knowledge of the green turtle life cycle at a high-latitude site; they demonstrate that green turtles show fidelity to distinct use areas within developmental habitats over many years and exhibit seasonal movements.

## Introduction

Green turtles (*Chelonia mydas*) are long-lived, marine megaherbivores exhibiting extensive movements across ocean basins during their lifetimes but also showing remarkable site fidelity to specific sites. Early tagging studies showed fidelity to nesting beaches by reproductive females making long reproductive migrations from their foraging grounds [1–3]. Fidelity of both immatures and adults of both sexes to foraging grounds was also revealed by early tagging studies [2, 4]and later elucidated by satellite telemetry, *e*.*g*., Broderick et al. [5]; Shimada et al. [6] and acoustic telemetry (reviewed in Hardin and Fuentes [7]). Recent studies have observed fidelity of green turtles to resting and overwintering areas, *e*.*g*., Lamont et al. [8], Petit et al. [9]. Travel that follows hatching, during the oceanic or surface-pelagic stage, may take green turtles far from their natal beaches and, in many cases, far from the foraging areas they will use as adults. Immature turtles undertake developmental migrations that are likely to cover larger distances than the reproductive migrations that they will make as adults [10–12].

For green turtles, the surface-pelagic phase is spent at or near the surface in the open ocean where, at least in the Atlantic system, they are often associated with *Sargassum*-dominated surface-pelagic drift communities [13–16]. After a period estimated to be 3 to 5 years [17], they settle at foraging grounds where they feed on the bottom. This developmental transition involves both a change in diet and a change in feeding location within the water column; it is an abrupt transition from a mobile, surface-pelagic existence to far more fixed benthic behavior. During this second, benthic, developmental phase, that may last >20 years, green turtles have their first opportunity to establish fidelity to sites offering the resources they need.

Bermuda (32.3° N, 64.8° W) is one of many locations in the Atlantic system that provides benthic developmental habitat for green turtles [11] and is an ideal site to study the details of habitat use at this stage. It is located on an isolated shallow platform nearly 1,000 km from the nearest alternative benthic habitat for green turtles (North Carolina, US). The Bermuda Platform supports a mixed-stock foraging aggregation of immature individuals where no adults are present [11, 18, 19]. This provides an opportunity to examine the movements of benthic immature turtles without potential influence of adults.

The purpose of the present study was to characterize the range of habitats and behaviors used by immature green turtles while living on and departing from the Bermuda Platform. Data collected during this study complement the long-term mark-recapture study of immature green turtles in Bermuda (the Bermuda Turtle Project, hereafter BTP). Argos satellite telemetry had been used by BTP previously to confirm the site fidelity that was indicated by mark-recapture data [11]. However, that technology did not allow study of movements and fine-scale habitat use. The higher-resolution location data that can now be generated by Fastloc-GPS-enabled transmitters, combined with information on habitat, temperature, and depth, can be used to better understand local movements, patterns of habitat use, and site fidelity. At this study area at the northernmost site where green turtles are year-round residents in the North Atlantic [20–22] the data are particularly useful for examining responses to seasonality in water temperatures. The Fastloc-GPS technology also allows the documentation of precise travel paths and travel behavior of green turtles as they emigrate from this developmental aggregation. Understanding exactly which habitats green turtles use on the Bermuda Platform, how and where they spend their time while on the Platform, and where their migrations take them when they depart have both local and global conservation management value.

## Methods

**Fig 1:**
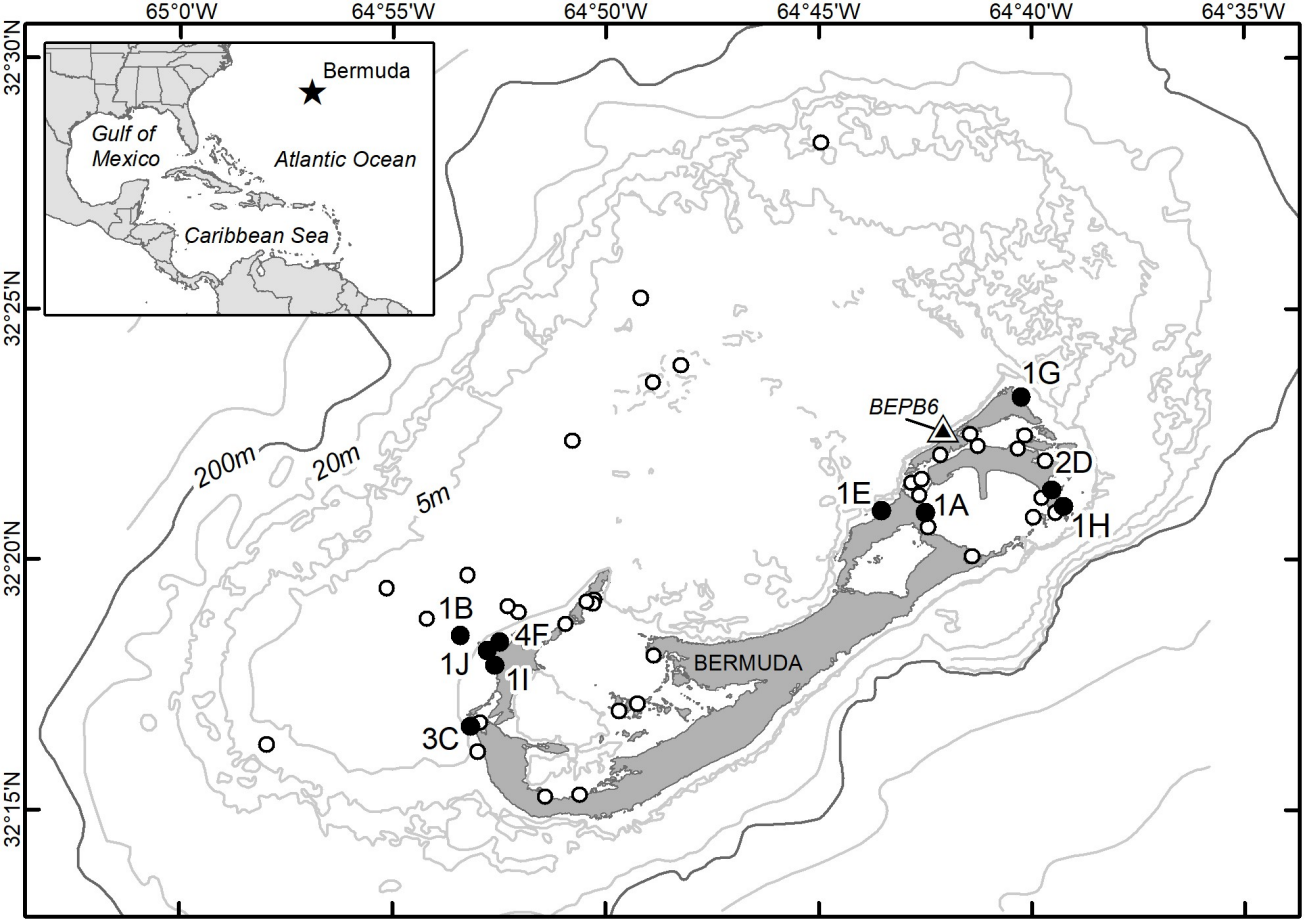
Bermuda Turtle Project netting sites (circles). Sites at which satellite transmitters were deployed are represented by filled circles and the number of deployments per site is noted. The triangle denotes the location of NOAA National Data Buoy Center station BEPB6-2695540 (http://www.ndbc.noaa.gov/station_page.php?station=BEPB6) from which we obtained water temperature data. Bathymetric contours provided by the Bermuda Department of Environment and Natural Resources; selected contours are labeled (depth in m).

We deployed satellite transmitters on 16 immature green turtles captured in Bermuda from 2011–2018 as part of ongoing studies [11, 19]. Turtles were captured using an entrapment net (427–610 × 6 m) set in a closed ring for 1–2-h intervals on seagrass meadows at predetermined study sites around the Bermuda Platform (Fig 1). A team of snorkelers continuously searched the nets during deployment, promptly removed entangled turtles, and placed them in a support vessel. All turtles were tagged on the trailing edge of both foreflippers using monel, inconel, or titanium tags; a passive integrated transponder (PIT) tag was placed in the left front flipper between the radius and ulna. We collected standard morphometric data and biological samples for related studies from all captures. Turtles were selected for satellite tracking based on adequate size to bear the transmitter [23] and good physical condition. Turtle size was evaluated as minimum straight carapace length in cm measured from the nuchal notch to the pygal notch (SCLmin). We tracked turtles captured at study sites across a diversity of sampling sites around the Bermuda Platform.

Transmitters were Wildlife Computers SPLASH10-F-400 (4) and SPLASH10-F-296 (12) tags capable of providing Argos and Fastloc-GPS positional data. All units were programmed using Wildlife Computers Mk10Host software to transmit data via the Argos system following a 4 h on/2 h off duty cycle beginning at 0 h GMT. Fastloc-GPS data were collected at 2h intervals during even hours (GMT). Dive depth and water temperature data were collected during 14 of the 16 deployments. Transmitter depth sensor resolution was 0.5 m and dives less than 1 m were ignored. The 14 dive depth bins were set to 1, 2, 3, 4, 5, 10, 12, 14, 16, 18, 20, 25, 30, >30 m for the initial two deployments and 1, 2, 3, 4, 5, 7, 9, 11, 13, 15, 15, 17, 19, 24, 29, >29 m for all transmitter deployments. Water temperature bins were set to 12, 13, 14, 15, 16, 17, 18, 19, 20, 22, 24, 26, 28, >28 degrees Celsius for four deployments and 15, 16, 17, 18, 19, 20, 21, 22, 23, 24, 27, 30, 33, >33 for the latter 10 transmitter deployments. We summarized binned dive data by calculating means weighted by the proportion of time the turtle spent in each bin. Bermuda is on Atlantic Standard Time (AST).

We attached transmitters with a slow-curing, two-part epoxy (Powers^™^) using methods similar to those described by Mansfield [24]. Transmitters were attached to the highest point on the carapace. The attachment site was cleaned of epibiota, lightly sanded, and dried with isopropanol. We initially dispensed mixed epoxy into a disposable container until the epoxy mix appeared uniform. The epoxy in this container was used to monitor the curing temperature; when the container became hot to the touch, we cooled the epoxy on the turtle’s carapace using freshwater rinses. Transmitters were embedded in a thin layer of epoxy with additional epoxy added around the base. Once the first layer of epoxy had set, we applied an additional layer of two-part epoxy putty (Sonic Weld^™^) around the edge of the units and smoothed it, creating a hydrodynamic shape following the curvature of the carapace to reduce the frontal area of the tag, thus reducing drag and minimizing the likelihood of detachment due to impacts from rocky substrates [25]. The hardened epoxy and transmitter were painted with an ablative marine antifouling paint (Petit^™^). Telemetered turtles were released at their capture location, typically within 2–3 h of capture. Two individuals (platform transmitter terminals [PTTs] 120326 and 132093) were held overnight and released the following morning as part of “Tour de Turtles,” a public outreach and conservation education event conducted by the Sea Turtle Conservancy and the Bermuda Zoological Society. In these two cases, a turtle from the last net set of the day was selected to reduce the hold time to approximately 12 h. Individuals were housed in a large tank provided by the Bermuda Aquarium which was filled with fresh seawater. Turtles were released the following morning from shore and < 1 km from their capture site.

Raw Argos location estimates were subjected to plausibility filtering using the Douglas Argos Filter Algorithm (DAF) [26], implemented using SAS Enterprise Guide 7.1 (SAS Institute, Inc. Cary, NC USA). We set the filter parameters to values consistent with other local-scale analyses of sea turtle tracking data, *e*.*g*., Foley et al. [27]. Argos locations were used only to confirm the general location of tracked turtles during instances of prolonged gaps in Fastloc-GPS positions. We used only Fastloc-GPS positions for all analyses.

Argos messages were downloaded and processed using the manufacturer’s software (Wildlife Computers Data Analysis Program) to obtain Fastloc-GPS positions, depth, and water temperature data. We evaluated the plausibility of Fastloc-GPS positions using customized data-processing routines written in R (R Development Core Team 2019) that assessed travel path metrics and the quality of Fastloc-GPS position solutions. A Fastloc-GPS position solution was considered potentially errant if 1) the number of satellites used in the position solution was fewer than 6 and 2) the residual error associated with the solution exceeded 30 [28]. We expected the exclusion of these potentially errant locations to result in a dataset with positional accuracy of < 100 m [29, 30]. We evaluated the data provided by each transmitter to determine whether the cessation of transmissions was due to battery exhaustion, saltwater switch malfunction (potentially caused by biofouling), epoxy adhesion failure, or mortality [31–33].

We analyzed Fastloc-GPS positions collected by each transmitter in ArcGIS (ESRI, Redlands, CA) to identify spatially and temporally discrete concentrations of activity or habitat use. This process was facilitated by calculating the mean centers and densities of Fastloc-GPS positions at hourly (to identify diurnal or nocturnal patterns), monthly, and seasonal intervals. We examined dive depth and water temperature data at similar intervals. When possible, we broadly categorized benthic habitats in high-use areas for turtles based on qualitative habitat assessments made during approximately 50% of all sampling events (383 of 743 individual sets of the entrapment net) between 1992 and 2018 (see Meylan et al. [19]), along with opportunistic visits to nearby reef sites, and by features visible in satellite imagery (*e*.*g*., reef, seagrass meadow). We digitized vessel navigation channels near study sites using navigational buoy locations, nautical charts, and satellite imagery.

For each tracked turtle, we identified stationary phases of habitat use using the segmentation method developed by Patin et al. [29], implemented using the “segclust2d” R package. We used the segmentation method to identify distinct shifts in local movements (*e*.*g*., seasonal changes) and to determine when developmental migrations were initiated. This approach has been applied to green turtle movement data by Siegwalt et al. [30], and we implemented this method in a similar fashion. All Fastloc-GPS data for each turtle were first screened by the data-driven SDLfilter implemented in R [32, 33]. Fastloc-GPS locations were then temporally re-discretized at a 12-h time interval using the “adehabitat” R package [34]. Data from each individual were then segmented into two or more phases with a minimum phase duration of 20 days. Phases shorter than 20 days or those containing positional data gaps of more than 3 days were not considered distinct phases of habitat use. We used Bhattacharyya’s coefficient (BC) to evaluate the degree of similarity of 95% utilization distributions for each phase identified by the segmentation analysis; this step was implemented using the “adehabitat” Kernel Overlap function. Phases with BC scores <0.5 were considered distinct periods of habitat use. For four turtles that emigrated from Bermuda, points associated with departure were not included in analyses of total residence areas. For turtles that departed, we calculated travel rate metrics and associated surface circulation velocity values using the Hybrid Coordinate Ocean Model + Navy Coupled Ocean Data Assimilation Global 1/12° dataset accessed via the Marine Geospatial Ecology Tools for ArcGIS [35].

We refer to the area encompassing all localized movements, such as foraging, resting, and transits between those, as the individual’s total residence area (minimum convex polygon, MCP). We included seasonally used habitats such as thermal refugia as part of the residence area. Thus, the total residence area includes all Bermuda Platform locations used by tracked individuals. It does not include directed travel associated with long-distance migrations away from the Bermuda Platform or short forays that preceded developmental migrations that were distinct from total residence area behaviors.

Using only Fastloc-GPS locations, we estimated total residence area (MCP) and three measures of home range (25, 50, and 90% utilization distribution, UD) using the kernel home range method implemented within the adehabitatHR. The 50 and 90% UDs have been used previously to represent the “core” and “overall” residence area of sea turtles, respectively, e.g., Foley et al. [27]. We use the term residence area in this paper in the sense of “total residence area” (MCP). We examined plots of the cumulative MCP area against deployment duration to determine if the total residence area had reached an asymptote.

To calculate home range estimates, we applied the rule-based ad hoc method for selecting the kernel smoothing parameter following Kie [36] to minimize over- or under-smoothing. We began the iterative process with an ad-hoc smoothing parameter, then gradually reduced the parameter’s value and stopped at the smallest value that produced a continuous 95% UD. This method has previously been applied to green turtle satellite telemetry data [37]. For 15 of 16 turtles, we excluded the first 24 h of location data from total residence area analyses and for all 16, any potentially errant locations and any locations associated with directed travel away from Bermuda. One individual, PTT 151800, did not return to the initial capture site upon release but instead moved ~16 km west and then remained within a typical-size 90% UD at this site for a 40-day period. Thus, data from that residence period were used in segmentation and UD analyses but not the directed travel to that area. Lastly, we visually examined Fastloc-GPS positions within each turtle’s total residence area to identify discrete clusters of locations which represented concentrations of activity or repeated use of distinct habitats. We classified these “distinct use areas” into foraging and resting areas based on habitat and turtle movement data characteristics. We defined cool weather refugia as areas that were distinct from seagrass meadow foraging or resting habitats and were visited only during cool months. We manually digitized these distinct use areas within ArcGIS to estimate their size, average water depth, and timing of use (e.g., day, night, seasonal). We calculated the geographic mean center of each area and used that position to measure the distance among different areas of concentrated habitat use for each turtle. Water depth data for the seagrass meadows were obtained from depth measurements collected using hand-held sonar devices during the project’s capture sessions. Water depths for other portions of the turtle’s residence areas were obtained from the 1 arc-second Bermuda digital elevation model (NOAA NCEI [38]).

**Fig 2:**
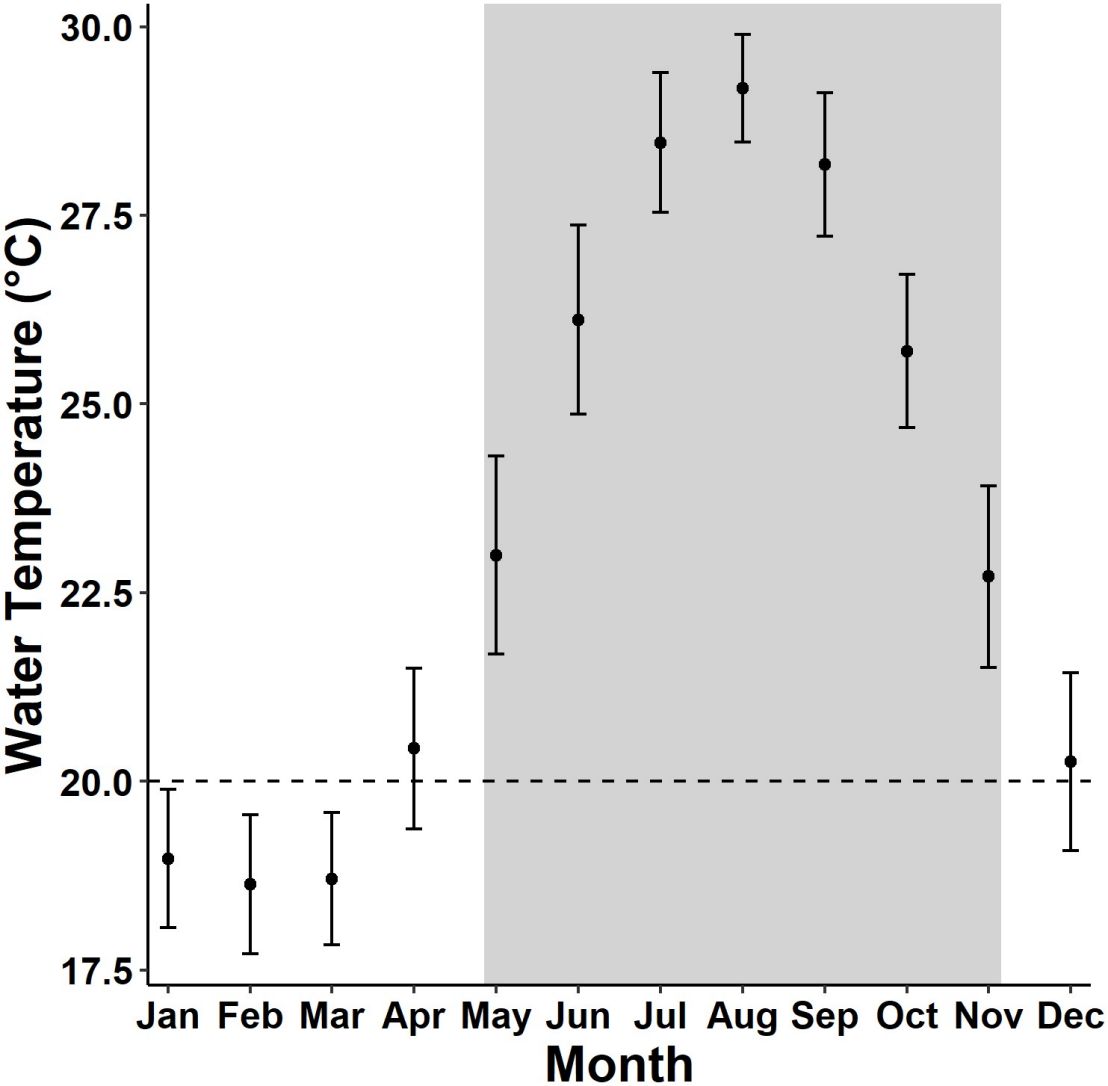
Monthly water temperatures Jan 2009–Nov 2019, NOAA National Data Buoy Center station BEPB6-2695540, Bermuda. Error bars represent the standard deviation around the mean monthly water temperature. The horizontal dashed line represents the 20°C threshold we used to delineate cool and warm months (shaded period) in the analyses.

**Fig 3:**
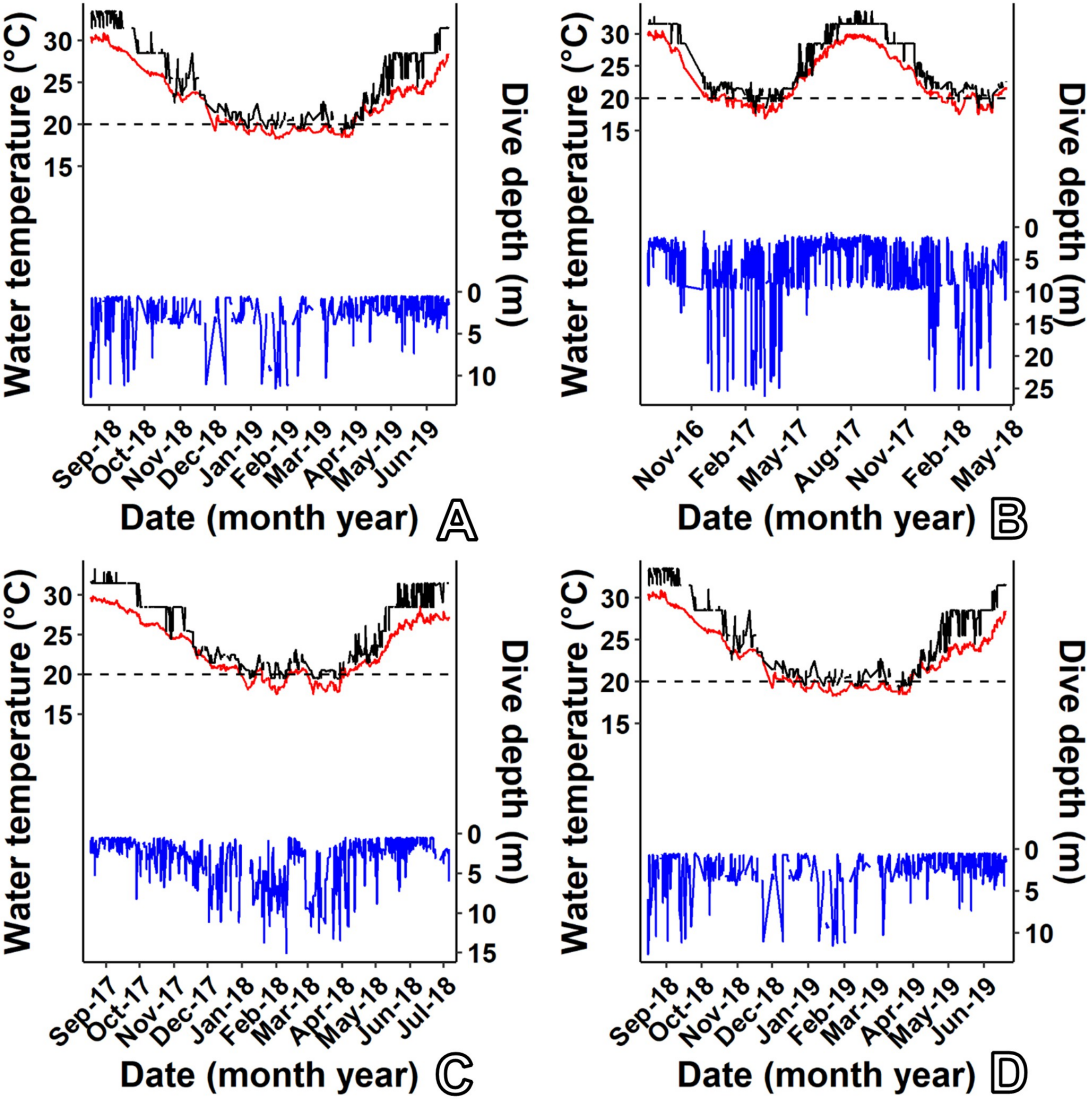
Dive profiles (blue line) for four C. mydas from Bermuda relative to ambient water temperature as recorded by transmitters (black line) and Data Buoy Center station BEPB6-2695540 (red line). Data are shown for (A) PTT 163692, (B) PTT 163693, (C) PTT 172208, (D) PTT 172209.

We obtained water temperature data from NOAA National Data Buoy Center station (BEPB6-2695540, URL: http://www.ndbc.noaa.gov/station_page.php?station=BEPB6) located on St. George Island (32.374° N, -64.701°W), Bermuda. We downloaded data from Jan 2009–Nov 2019, an approximately 10-year period bracketing the period of transmitter deployments (data for Dec 2019 were unavailable from this station). This station provided water temperature data collected at 6-minute intervals from a depth of 2.8 m below mean lower low water, a depth similar to that of our study sites. We used these data as a proxy for water temperature at the seagrass beds where the transmitters were deployed. We acknowledge that actual temperatures at our study sites may have varied from those reported by this station because of physiographic factors; however, no other continuous water temperature data for our 9-year study period were available. Data from the BEPB6 station were used to identify seasonal temperature patterns that likely occurred across the Bermuda Platform. Data reported by the transmitter temperature sensors were used as a measure of the actual temperature experienced by the telemetered turtles and were evaluated against temperature data obtained from the BEPB6 station. We summarized BEPB6 water temperatures by month and defined a “warm” season from May–Nov and a “cool” season from Dec–April based on when temperatures tended to be above 20°C (Fig 2) and on our observations of distinct changes in dive behavior of some turtles at this temperature (Fig 3).

**Fig 4:**
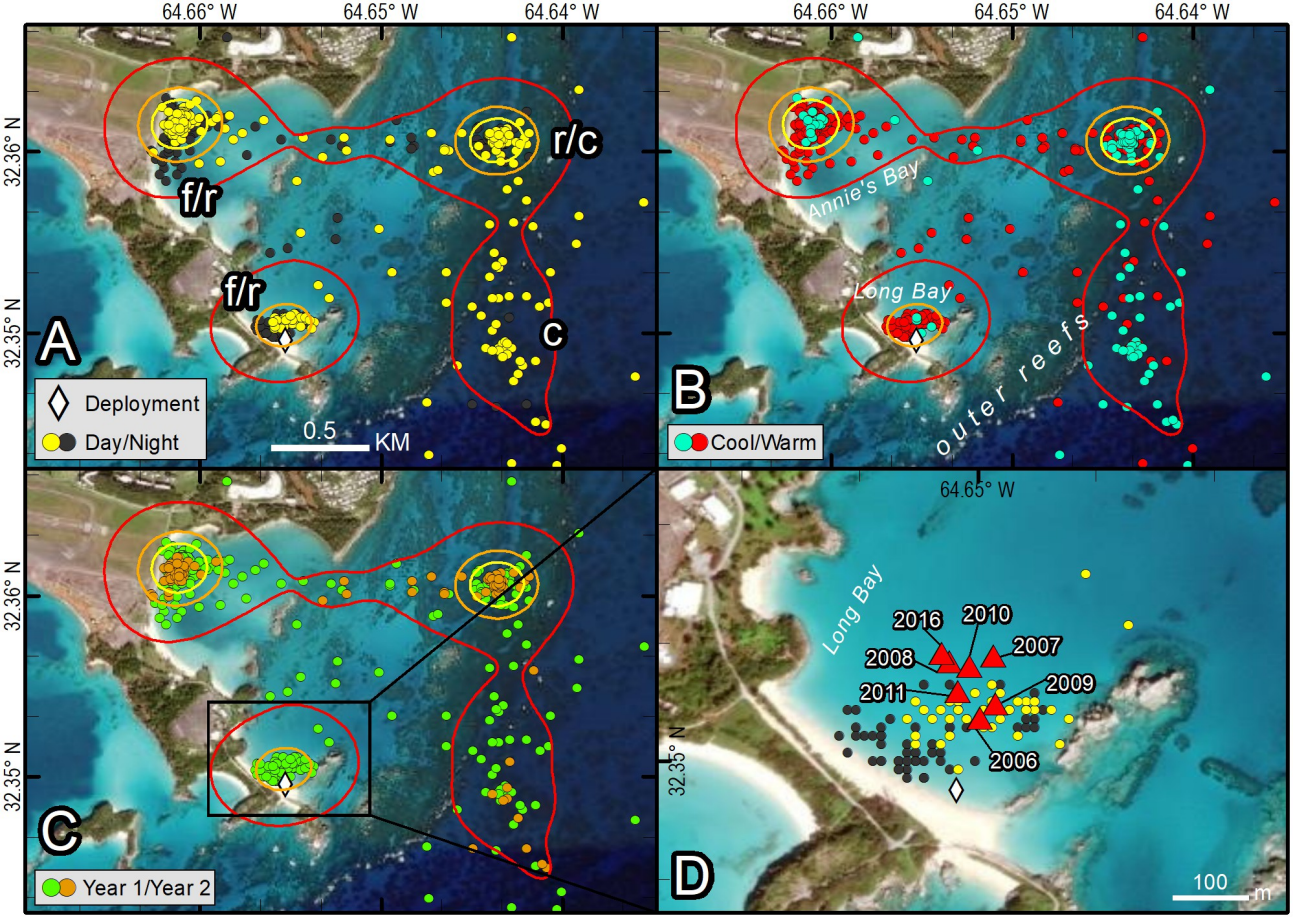
Fastloc-GPS locations (circles) and home range utilization distributions (25%—yellow, 50%—orange, and 90%—red polygons) of PTT 163693 on an immature green turtle (*Chelonia mydas*), Long Bay, Bermuda, Aug 18 2016–April 27 2018. Three distinct use areas were revealed: the primary foraging and resting area used during all months and during daytime and nighttime hours (f/r), the resting and cool weather refugia used during all months and during daytime and nighttime hours (r/c), and a cool weather refugia used primarily during daylight hours Nov 2016–May 2017 and Nov 2017–Feb 2018 (c). Locations are coded for photoperiod (A), season (B), and year that locations were received (C). Panel D shows detail for the initial foraging and resting area used by this individual and includes locations of all net captures (triangles). Republished from Esri, DigitalGlobe, GeoEye, Earthstar Geographics, CNES/Airbus DS, USDA, USGS, AeroGRID, IGN, and the GIS User Community under a CC BY license, with permission from Esri, original copyright 2022.

We mapped location data for each transmitter and reviewed movements during daytime and nighttime, and during warm and cool months (e.g., Fig 4). For each tracked turtle, we examined correlations between the distance from each Fastloc-GPS location to the mean center of the 25% kernel UD within the residence area and two potentially explanatory variables, depth and seawater temperature. Correlations were examined using Pearson’s product-moment correlation coefficient implemented in R. All means are reported ± one standard deviation.

For turtles that exhibited long-distance directed travel (i.e., developmental migration), we calculated travel rate, bearing, and path straightness. We calculated path straightness using a 3-position straightness index based on the straightness index defined by Batschelet [39]. The index compares the distance traveled along subsets of three sequential positions (A, B, and C) to the distance between the first and last position (straight line from A to C). The overall straightness was derived from this process which was iterated over the path positions. We also attributed sea surface current velocity values to migratory positions using the Marine Geospatial Ecology Tools for ArcGIS [35].

We used tracking data from 11 of 16 transmitters to estimate the detection rate of the entrapment net sampling method used by the BTP since 1976. Detectability was estimated for individuals captured at the deployment site one or more times before or after tracking. We assumed that the site fidelity behavior illustrated by tracking data had remained constant between net captures. Dividing the total number of net captures by the number of sets of the entrapment net within the presumed foraging area for that individual during the total residency period provided an estimate of detectability. We restricted this estimate to individuals with a total residency period of more than one year. Individuals that were known to have moved between separate seagrass meadow sampling sites during their residency in Bermuda were excluded because it was not possible to know the number of sets of the entrapment net that occurred where that individual could be assumed to be resident.

### Ethics statement

We followed all applicable guidelines for handling live sea turtles and obtained all necessary approvals.

Sea turtle capture, data collection, and satellite transmitter application in Bermuda were authorized under a series of written agreements and permits from the Bermuda Department of the Environment and Natural Resources, most recently, License No. 2018071309. No IACUC agreement was required for this work, however, the same methods are used by PAM and ABM in Panama and have been approved by the Smithsonian Tropical Research IACUC committee, most recently STRI ACUC 2020–0414–2023.

## Results

### Deployment summary

We deployed transmitters on 16 immature green turtles ranging in size from 48.4 to 69.4 cm SCLmin (mean 62.3 ±5.0 SD, Table 1). Deployments were made at 10 different localities on the Bermuda Platform (Fig 1), always beginning in the month of Aug, during the years 2011–2018. Transmitters operated for an average of 284 days (±215 SD, range 37–779) during which we received data on an average of 220 days (±164 SD, range 38–591); we received an average of 562 (±515 SD, range: 174–2,007) Fastloc-GPS locations per individual (Table 1).

**Table 1:** Platform terminal transmitters (PTT) deployed on immature green turtles (*Chelonia mydas*) in Bermuda, 2011–2018. Carapace length (SCL_min_, cm), deployment site on the Bermuda Platform, and tag longevity details are provided for each deployment. Turtles denoted by the superscript “D” were observed making developmental migrations away from Bermuda. Letters following site names correspond to labels in Fig 1. Minimum convex polygons (MCP) and home range estimates (kernel utilization distribution of 25, 50, and 90%) (km^2^) are given for each tracked turtle.

PTT	SCLmin (cm)	Site	Date of deployment	Days deployed (Data days)	Fastloc-GPS locations received	Kernel UD			
						25%	50%	90%	MCP
108507	60.6	Blue Hole (A)	3-Aug-11	359 (194)	478	0.01	0.03	0.19	1.19
108508	48.4	Vixen (B)	11-Aug-11	315 (180)	207	0.08	0.20	0.71	1.20
120325	59.7	Wreck Hill (C)	10-Aug-12	289 (217)	490	0.03	0.08	0.39	1.13
120326	66.2	Annie’s Bay (D)	14-Aug-12	37 (38)	181	0.01	0.03	0.11	0.29
132092	69.4	Baileys Bay (E)	7-Aug-13	96 (97)	501	0.03	0.09	0.42	2.24
132093	68	Annie’s Bay (D)	13-Aug-13	66 (67)	252	0.29	0.68	2.39	5.39
140712*^D^	66.8	Somerset Long Bay (F)	8-Aug-14	779 (304)	1,641	0.06	0.17	0.90	2.62
140713*^D^	63.9	Wreck Hill (C)	11-Aug-14	504 (485)	306	0.10	0.25	0.92	0.52
140713*^D^ ^1^						0.37	0.96	4.45	2.42
151800	61	Ft. St. Catherine (G)	14-Aug-15	58 (59)	303	0.13	0.41	2.29	2.73
151801^**D**^	66.6	Wreck Hill (C)	17-Aug-15	117 (41)	355	0.23	0.63	2.55	0.44
163691†^**D**^	67.1	Somerset Long Bay (F)	10-Aug-16	50 (51)	591	0.18	0.58	3.05	2.81
163692†	58.5	Somerset Long Bay (F)	15-Aug-16	329 (321)	2,007	0.02	0.08	0.43	1.00
163693	57.2	Long Bay (H)	18-Aug-16	616 (591)	500	0.09	0.24	1.54	16.98
172208	59	Somerset Long Bay (F)	18-Aug-17	327 (318)	488	0.72	1.96	8.33	24.50
172209	65.7	King Charles Hole (I)	16-Aug-18	309 (281)	515	0.97	2.40	9.01	45.75
174108	59.2	Methelin Bay (J)	20-Aug-18	291 (281)	174	0.04	0.11	0.41	2.52
Mean						0.19	0.50	2.11	7.60

*Transmitter did not have depth sensor.

^†^Transmitter recovered after deployment.

^1^ The additional line for PTT 140713 is for home range established after developmental migration to North Bimini, Bahamas (see text) and is not included in the calculation of the mean.

### Initiation of track

All telemetered turtles were released on or adjacent to the seagrass meadow where they were captured. All turtles immediately swam to nearby deeper waters following release. All but one turtle returned to the meadow where they had been captured within approximately 24h; the one exception moved to a seagrass meadow 16 km to the west. Data from two recovered PTTs provided a higher level of detail on the turtles’ behavior during the initial week of deployment (S1 Fig). Both of these individuals returned to the seagrass meadow where they were captured and began daily transits between presumed foraging and resting areas after approximately 24 h. Because of the likelihood that capture and transmitter attachment had a short-term impact on turtle behavior, we excluded the first 24h of data from total residence area size estimations for all individuals. We observed no differences in the post-release behavior of the two individuals that were held overnight and released the following morning compared to other tracked turtles.

### Home range

**Fig 5:**
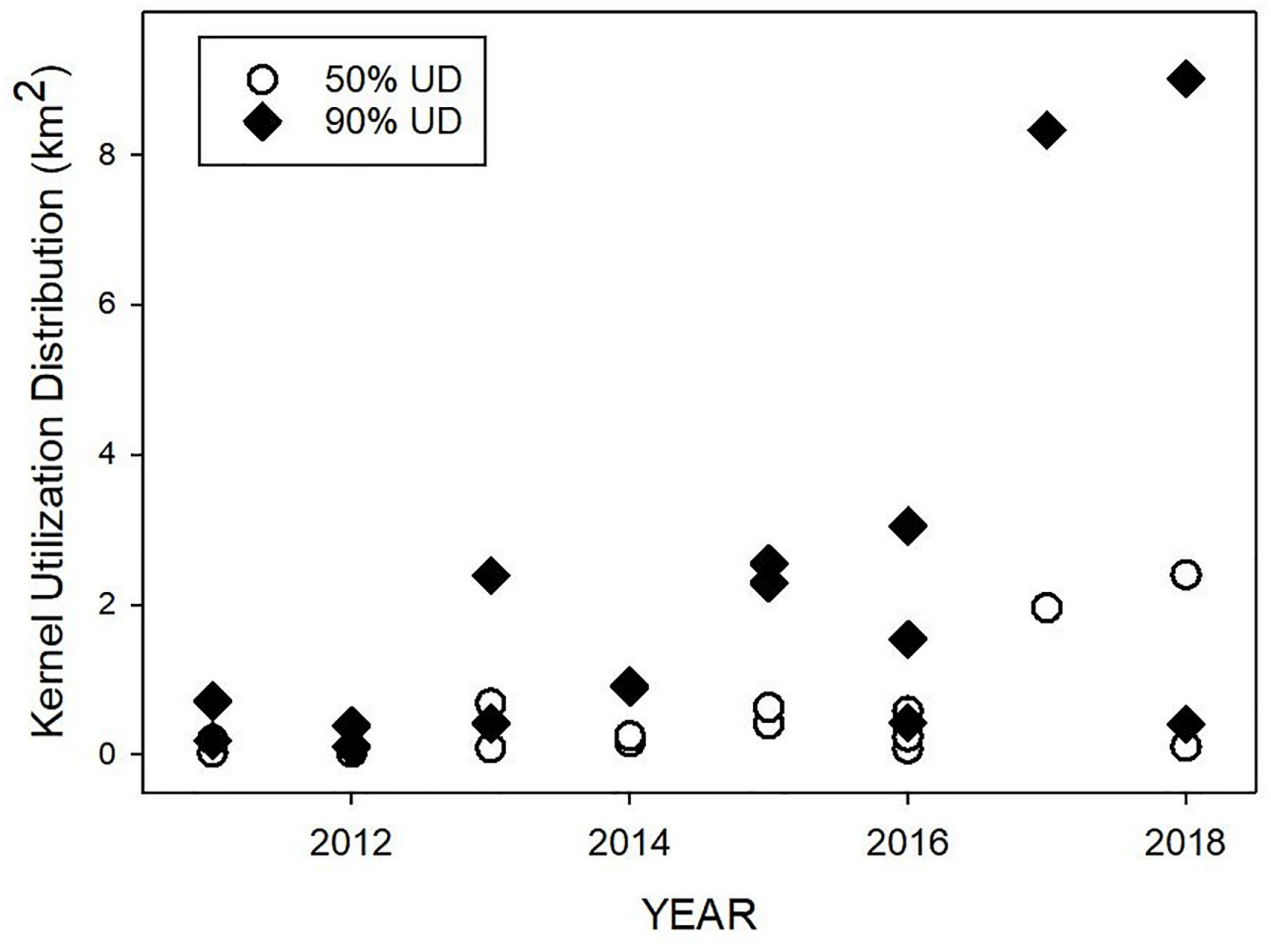
Size of fixed kernel utilization distribution (FKUD, 50%, 90%) estimates of home ranges for immature green turtle (*Chelonia mydas*) tracked in Bermuda by year of deployment (2011–2018). Open circles represent the 50% FKUD for each turtle. Filled diamonds represent the 90% FKUD for each turtle.

The MCPs of all tracked turtles reached an asymptote during the tracking period. The size of 25, 50, and 90% UDs and a MCP for each of 16 individuals are given in Table 2. Home range, estimated as 90% UD, varied in size from 0.11–9.01 km2 (mean 2.29 ±2.71 SD); core residence areas (50% UD) varied from 0.03–2.40 km2 (mean 0.54 ±0.69 SD). We did not find a relationship between the size of the 90% UD and turtle size, days deployed, data days, or quality of Fastloc-GPS positions (mean number of satellites per location). We did, however, detect a significant increase over the 8 years of the study, from less than 1 km2 before 2013 to 3 or more km2 in 2018 (R2 = 0.51, F1,14 = 14.55, p = 0.0019, Fig 5). Three individuals tracked toward the end of the study had MCPs that were, on average, an order of magnitude larger than those of all other tracked turtles.

**Table 2:** Distinct use areas within an individual turtle’s residence area. Mean depth (m) and size (km^2^) were estimated for discrete concentrations of locations: daytime concentrations on a seagrass meadow (f/r), daytime concentrations off of a seagrass meadow, nighttime concentrations off of a seagrass meadow (r), and seasonal (cool weather refugia) (c). Concentrations of activity that were not distinct were not included (*e*.*g*., overlapping foraging and resting areas). Bathymetry data did not adequately characterize seagrass meadow depth in some areas, for those cases we used the depth at the capture locations that was measured by BTP personnel (denoted with an asterisk). Individual PTTs listed twice represent turtles for which multiple distinct use areas of the same type were observed.

PTT	Daytime seagrass meadow (f/r)		Daytime off seagrass meadow (r)		Nighttime off seagrass meadow (r)		Cool weather refugia (c)	
	Depth (m)	Size (km^2^)	Depth (m)	Size (km^2^)	Depth (m)	Size (km^2^)	Depth (m)	Size (km^2^)
108507	2.4	0.034	9.8	0.017				
108508	2.9*	0.108	9.3	0.004	9.7	0.0025		
120325	1.3	0.023	6.7	0.005	6.6	0.0095	10.0	0.487
120326	2.9*	0.033			1.6	0.0046		
132092	1.6	0.014	7.6	0.060	3.5	0.0560		
132093	2.4	0.032	5.3	0.011	4.8	0.0119		
140712a	3.0*	0.198	7.8	0.018	10.7	0.0101	9.6	0.667
140712b					11.5	0.0033	12.4	0.527
140713	1.7	0.053	9.5	0.019				
151800	3.4*	0.145						
151801	6.8	0.007						
163691a	3*	0.022						
163691b	8.4	0.003						
163692	3*	0.055			9.2	0.0039	10.1	0.042
163693a	2.1	0.022						
163693b	2.7	0.080						
172208	3*	0.088					10.5	0.014
172209a	3*	0.018			10.3	0.003		
172209b	8.4	0.049						
174108	3.0*	0.034			10.7	0.006	9.9	0.005
**Mean**	**3.4**	**0.05**	**8.0**	**0.02**	**7.9**	**0.01**	**10.4**	**0.29**

### Distinct use areas

**Fig 6:**
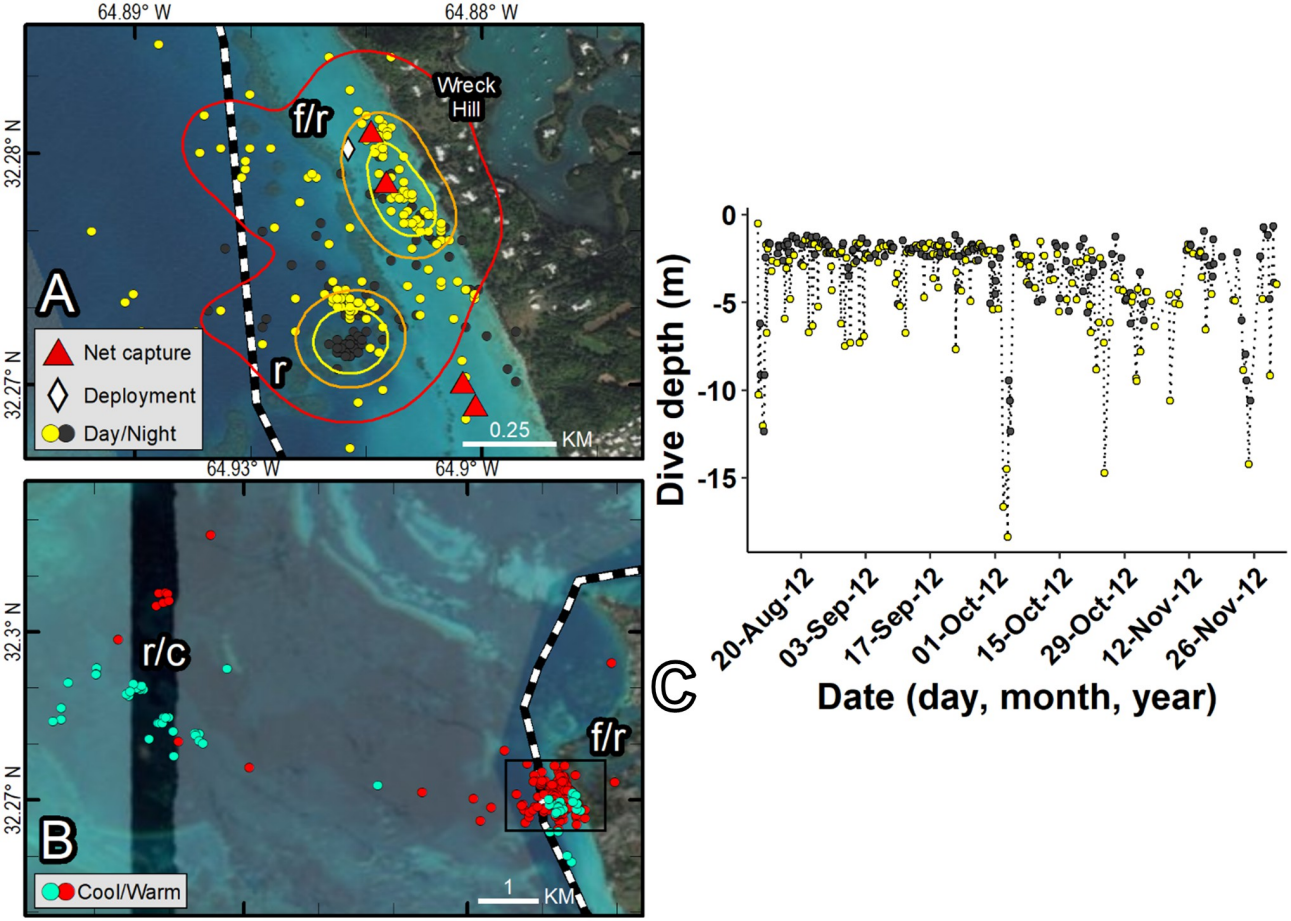
Fastloc-GPS locations (circles) and home range utilization distributions (25%—yellow, 50%—orange, and 90%—red polygons) of PTT 120325 on an immature green turtle (*Chelonia mydas*), Wreck Hill, Bermuda, Aug 10, 2012–May 27, 2013. Distinct use areas were revealed, including a presumed primary foraging and resting area (f/r) that was used during all months and during both daytime and nighttime hours, and a presumed resting and overwintering area used during all months and during both daytime and nighttime hours (r/c). Locations are coded for photoperiod (A, C) and season (B). Dashed line indicates primary boating channel (see Fig 4 for satellite image layer credits). C displays dive depth by photoperiod for warm month locations (red points) in (B) during day (yellow) and night (black). Republished from Esri, DigitalGlobe, GeoEye, Earthstar Geographics, CNES/Airbus DS, USDA, USGS, AeroGRID, IGN, and the GIS User Community under a CC BY license, with permission from Esri, original copyright 2022.

**Fig 7:**
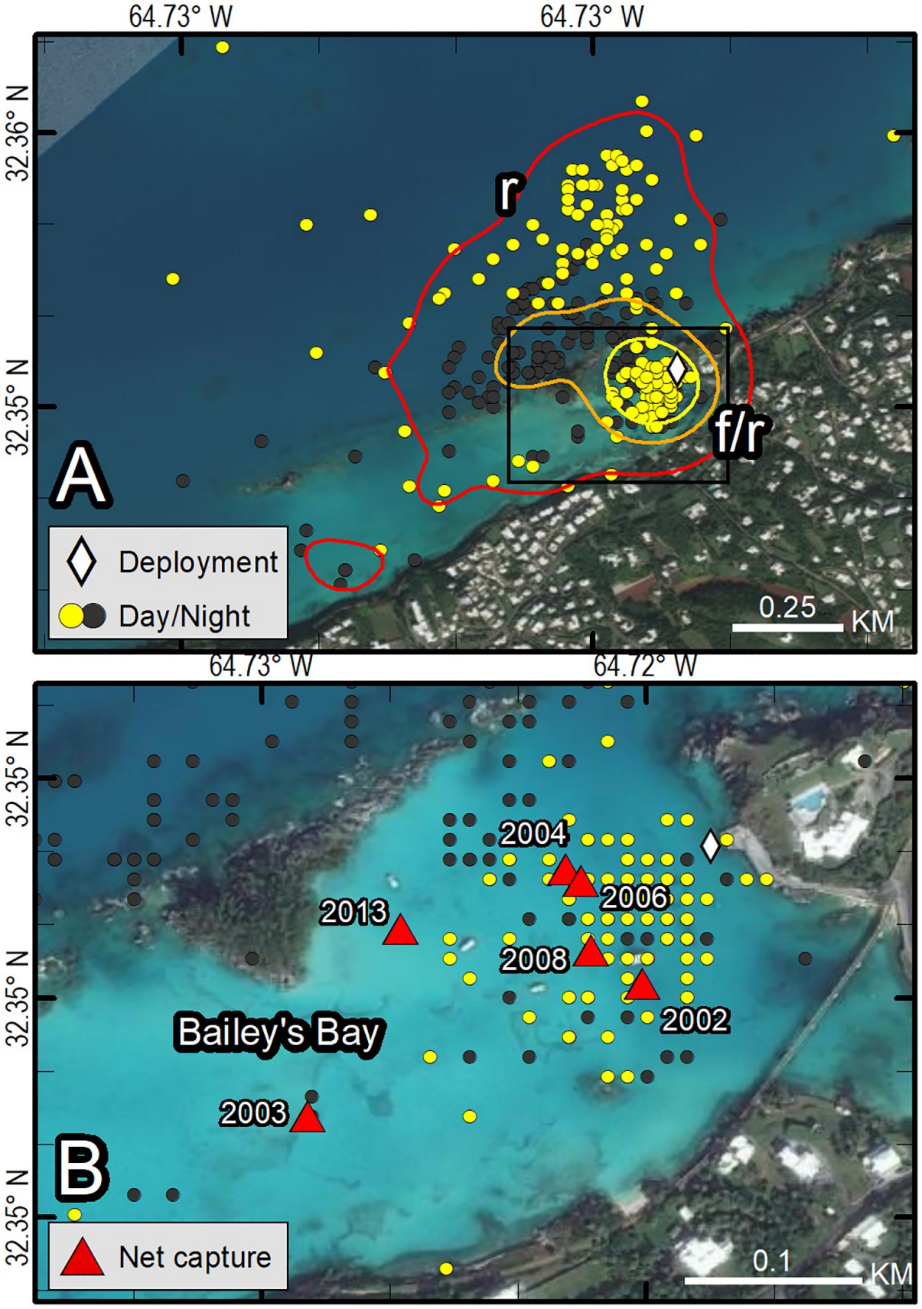
Fastloc-GPS locations (circles) and home range utilization distributions (25%—yellow, 50%—orange, and 90%—red polygons) of PTT 132092 on an immature green turtle (*Chelonia mydas*), Bailey’s Bay, Bermuda, Aug 7, 2013–Nov 11, 2013. Distinct use areas were revealed, including a presumed primary foraging and resting area (f/r) that was used during all months and during daytime and nighttime hours, and a presumed resting area away from the foraging area used during all months and during both daytime and nighttime hours (r). Locations are coded for photoperiod only. Republished from Esri, DigitalGlobe, GeoEye, Earthstar Geographics, CNES/Airbus DS, USDA, USGS, AeroGRID, IGN, and the GIS User Community under a CC BY license, with permission from Esri, original copyright 2022.

**Fig 8:**
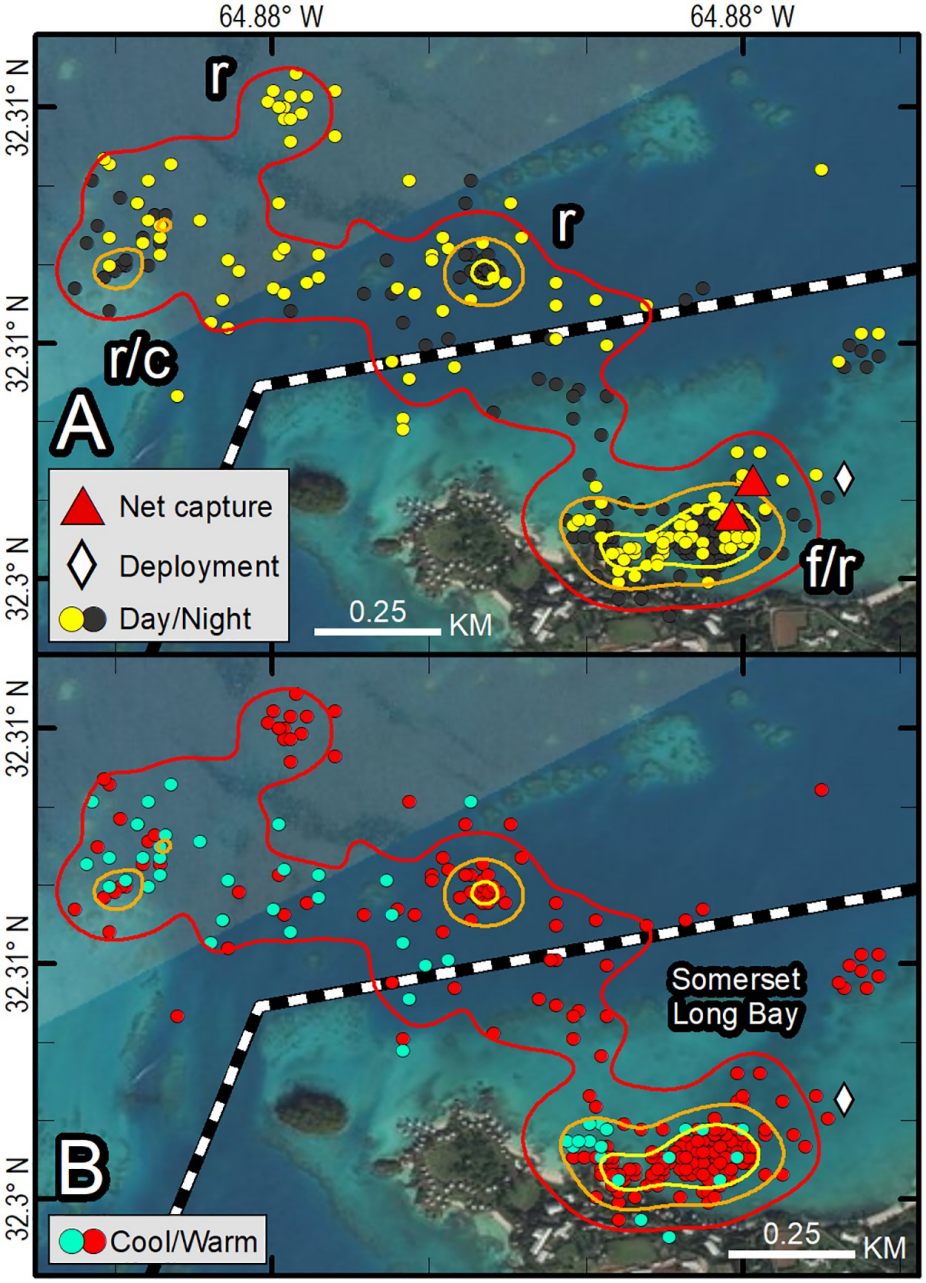
Fastloc-GPS locations (circles) and home range utilization distributions (25%—yellow, 50%—orange, and 90%—red polygons) of PTT 163692 on an immature green turtle (*Chelonia mydas*), Somerset Long Bay, Bermuda, Aug 15, 2016–July 10, 2017. Distinct use areas were revealed, including a presumed primary foraging and resting area (f/r) that was used during all months and during both daytime and nighttime hours, presumed resting areas away from the foraging area used during warm months (r), and a presumed resting and overwintering area away from the foraging area used during all months and during both daytime and nighttime hours (r/c). Locations are coded for photoperiod (A), season (B). Dashed line indicates primary boating channel. Republished from Esri, DigitalGlobe, GeoEye, Earthstar Geographics, CNES/Airbus DS, USDA, USGS, AeroGRID, IGN, and the GIS User Community under a CC BY license, with permission from Esri, original copyright 2022.

**Fig 9:**
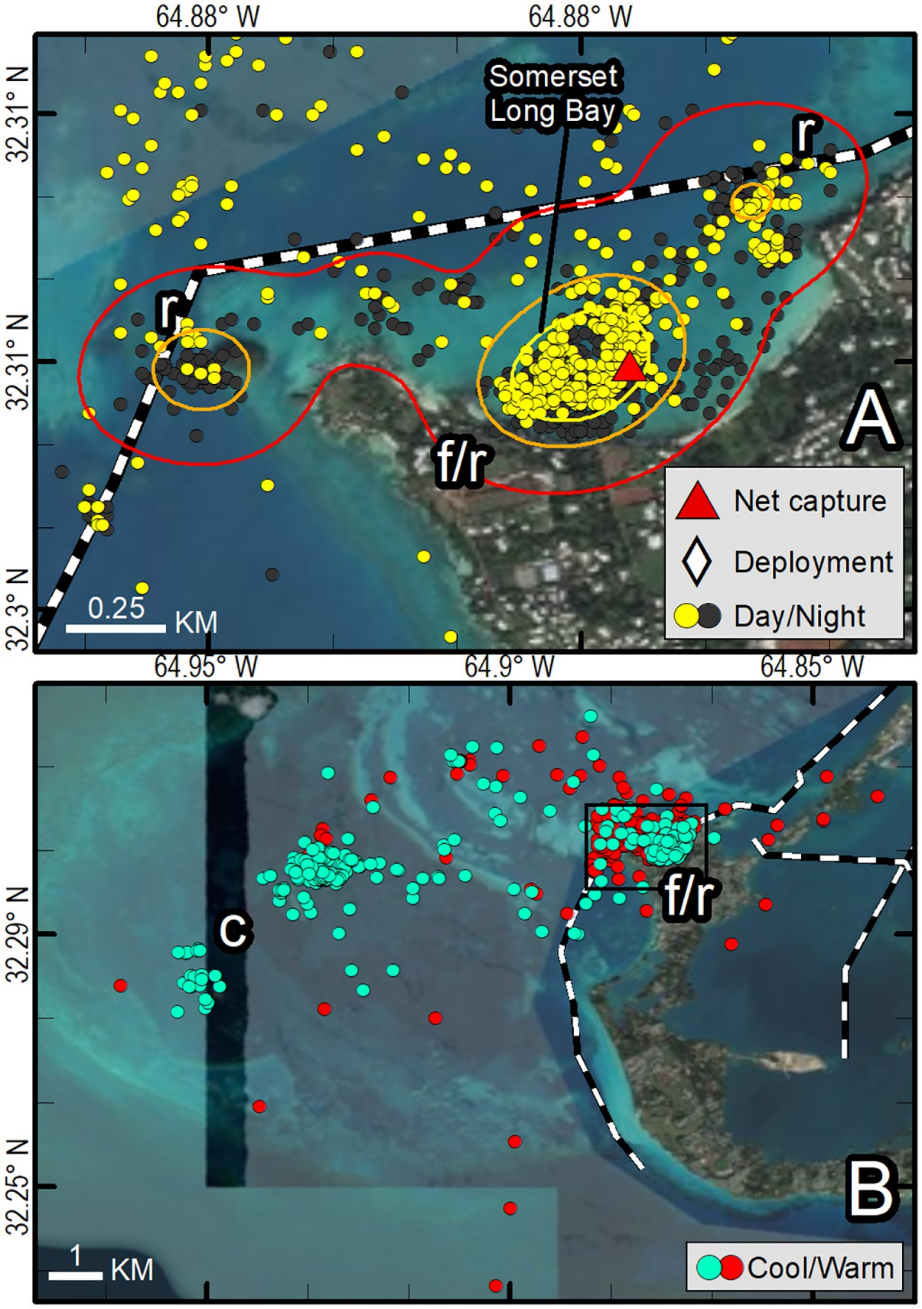
Fastloc-GPS locations (circles) and home range utilization distributions (25%—yellow, 50%—orange, and 90%—red polygons) of PTT 140712, Somerset Long Bay, Bermuda, Aug 8, 2014–Sept 25, 2016. Distinct use areas were revealed, including a presumed primary foraging and resting area (f/r) that was used during all months and during both daytime and nighttime hours, presumed resting areas away from the foraging area used during both warm and cool months (r), and a presumed cool weather refugia used during cool months (c). Locations are coded for photoperiod (A), season (B). Dashed lines indicate primary boating channels. Republished from Esri, DigitalGlobe, GeoEye, Earthstar Geographics, CNES/Airbus DS, USDA, USGS, AeroGRID, IGN, and the GIS User Community under a CC BY license, with permission from Esri, original copyright 2022.

All 16 turtles exhibited behavior consistent with usage of a residence area and, in most cases, concentrations of Fastloc-GPS positions within the residence area that we interpreted to represent “distinct use areas”. We identified the following types of distinct use areas: presumptive foraging areas (f), resting areas (r), and, for tracks with a sufficiently long record, cool weather refugia (c) as shown in Figs 4 and 6–9 (Table 2). We describe each type of distinct use area below.

#### Foraging areas (f)

**Fig 10:**
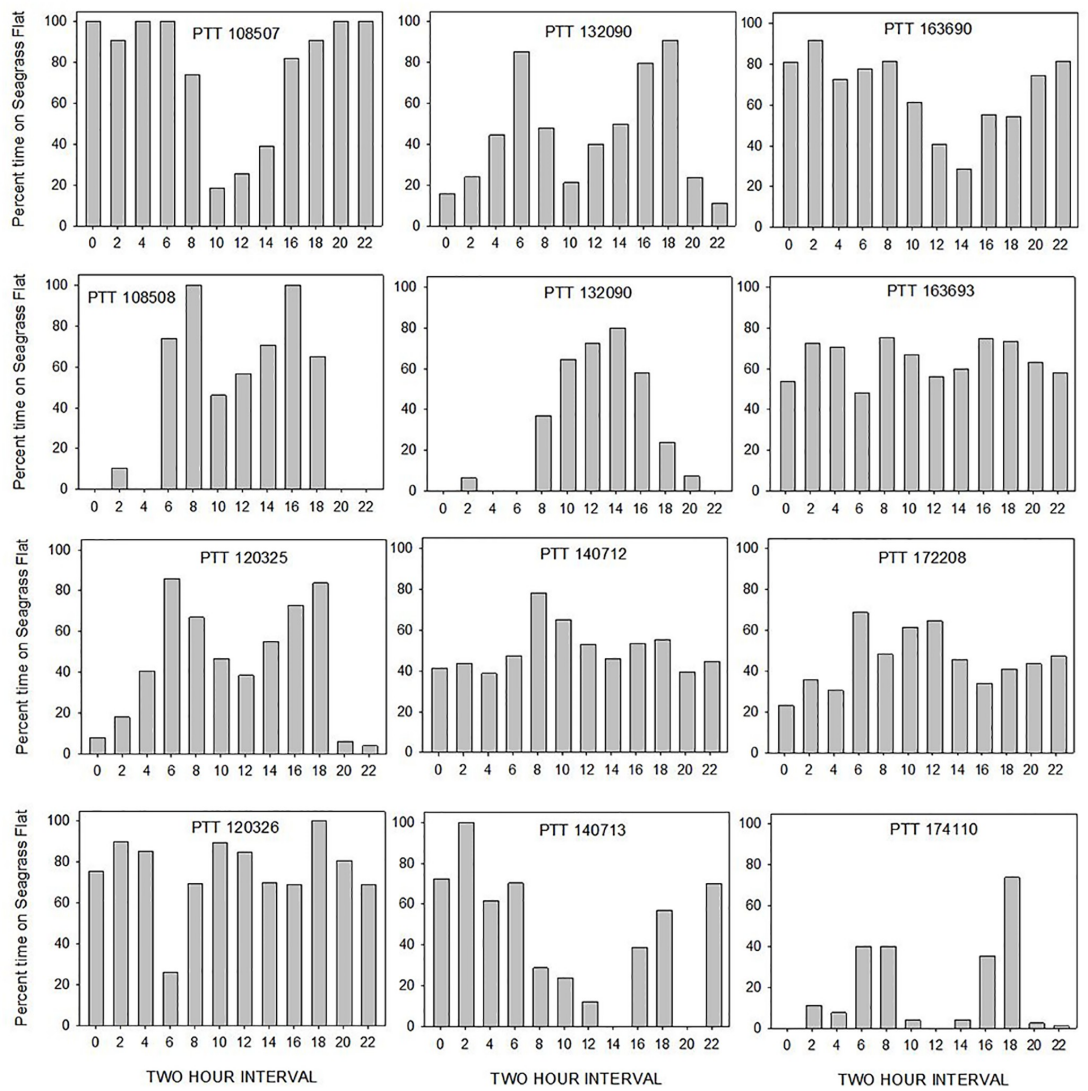
Pattern of daily use of Bermuda seagrass meadows during warm months (May–Nov) by individual green turtles (*Chelonia mydas*). Bars represent percent of Fastloc-GPS locations within seagrass meadows within 12 1-hour bins (even hours only, AST). Transmitters were programmed to provide locations during even hours (GMT).

Foraging areas were identified from concentrations of daytime positions that were distributed across one or multiple known seagrass meadows. During the period that transmitters were active, most turtles (13 of 16) showed site fidelity to a single foraging site and a single resting site away from the foraging area (9 of 16) within their residence area (e.g., Fig 6A). Three individuals used two discrete feeding areas (e.g., Fig 4A, Table 3). The seagrass meadow foraging concentrations (f) encompassed an average area of 0.05 km2 and had an average depth of 3.4 m (Table 2). We identified a pattern of bimodal daytime use of foraging sites during the warm season for 7 individuals (Fig 10) with peaks in use during morning (0600–0800 AST) and evening hours (1800–2000 AST). Tracked turtles typically departed foraging areas for daytime resting sites away from the foraging area during mid-morning and were least likely to be present on the seagrass meadow during mid-day hours (1000–1400 AST).

**Table 3:** Correlations of distance moved (from the mean center of the 25% kernel utilization distribution [UD]), and dive depth, with nearshore water temperature for 12 tracked green turtles (*Chelonia mydas*) on the Bermuda Platform, using Pearson’s product moment correlation coefficient.

	Correlation between temperature and distance moved		Correlation between temperature and dive depth	
PTT	Estimate	N	Estimate	N
108507	-0.59*	92	-0.29*	245
108508	-0.21	65	-0.45*	224
120325	-0.60*	119	-0.42*	420
132092	-0.18*	119	*Aug-Nov only*	
132093	-0.38	17	*Aug-Oct only*	
140712	-0.54*	268	*No depth sensor*	
140713	0.24	14	*No depth sensor*	
163692	-0.60*	50	-0.54*	692
163693	0.19*	133	-0.47*	1037
172208	-0.30*	122	-0.57*	725
172209	0.38*	65	-0.06	563
174108	-0.40*	81	-0.29*	531

Asterisks indicate significant correlation estimates (p < 0.05).

#### Resting areas (r)

**Fig 11:**
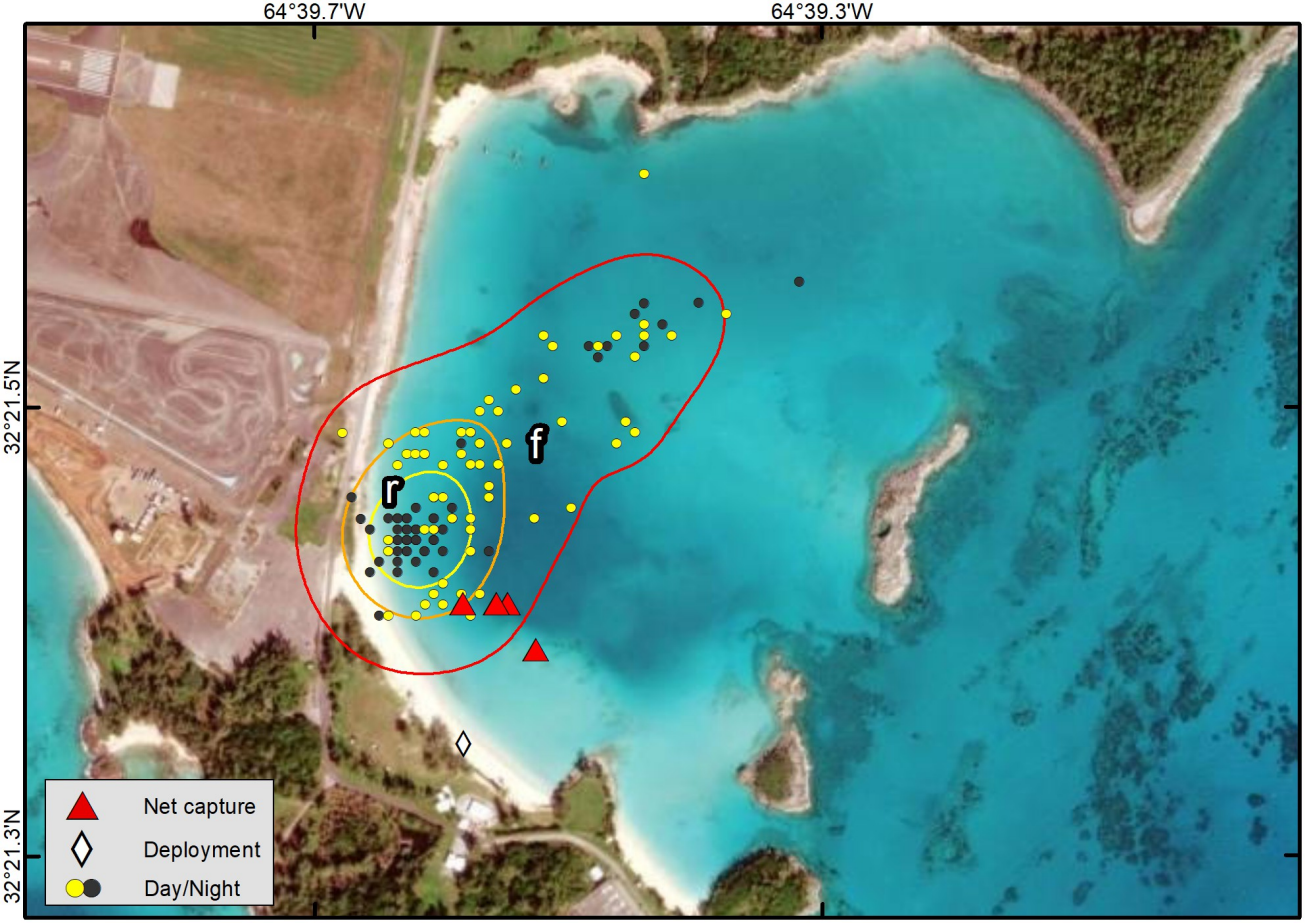
Fastloc-GPS locations (circles) and home range utilization distributions (25%—yellow, 50%—orange, and 90%—red polygons) of PTT 120326 on an immature green turtle (*Chelonia mydas*), Annie’s Bay, Bermuda, Aug 14, 2012–20 Sept 20, 2012. Distinct use areas were revealed, including a presumed primary foraging area (f), with a distinct nighttime resting area (r) within it, that was used throughout the tracking period. Locations are coded for photoperiod. Republished from Esri, DigitalGlobe, GeoEye, Earthstar Geographics, CNES/Airbus DS, USDA, USGS, AeroGRID, IGN, and the GIS User Community under a CC BY license, with permission from Esri, original copyright 2022.

Our data did not allow us to distinguish daytime or nighttime resting activity on the foraging ground from foraging activity, and therefore, daytime and nighttime points on known seagrass beds are labeled to reflect both activities (f/r). Daytime and nighttime resting areas away from the foraging area were identified from concentrations of positions that were spatially and temporally distinct from foraging areas. These areas were often located in deeper water at nearby reefs or rocky outcroppings in areas thought to have little or no seagrass. Turtle movements at these resting sites were constrained to smaller areas than those observed on the seagrass meadow foraging sites (Figs 6, 8 and 9; Table 2). Most turtles (11 of 16) made daily transits to these discrete areas that we classified as resting areas away from the foraging area. These presumed resting areas were typically used during mid-day (yellow points away from seagrass meadow) or nighttime hours (black points) (Figs 4 and 6–9). One individual used a distinct nighttime resting area on the seagrass meadow that was closer to shore than most presumed foraging positions (Fig 11). In most cases, location data from resting sites away from the foraging areas were concentrated (Figs 4A–4C and 6A), but for three turtles, Fastloc-GPS positions that were off of the seagrass meadow were dispersed and a single discrete resting area could not be identified (Figs 7A, 8A and 8B). We identified distinct daytime resting areas away from the foraging area for 7 of the tracked turtles, and distinct nighttime resting areas away from the foraging area for 8 of the tracked turtles; one of the latter used multiple nighttime resting areas (Table 1). Daytime and nighttime resting locations (r) away from the foraging sites were 0.02 km2 and 0.01 km2 in size on average, respectively, and occurred at average bathymetric depths of 8.0 and 7.6 m. During warm months, turtles spent an average of about 20% of daytime hours away from seagrass meadows at these resting sites which were 773 m apart on average (±389 SD, range: 334–1,270 m).

Nine of 16 turtles had one or two distinct areas for nighttime resting away from the foraging area (e.g., Figs 6A and 9A) but for others, Fastloc-GPS locations collected during nighttime hours sites were dispersed, preventing the identification of distinct nighttime resting areas (e.g., Figs 7A and 8A). Eight turtles spent some nights at the foraging site, shoreward of their daytime positions and in shallow water (e.g., Figs 4D, 9A and 10A). Five turtles rested away from the seagrass meadows during nighttime hours. During warm months, those individuals spent an average of approximately 80% of nighttime hours at resting sites away from the seagrass meadows. The average distance between foraging and those nighttime resting sites was 691 m (±382 SD, range: 163–1,294 m).

We identified distinct shifts in use areas for five individuals while they remained on the Platform, based on the segmentation analysis results. Four turtles exhibited shifts in areas used during fall and winter months compared to warmer months. For another (PTT 172209), segmentation analysis detected two use areas during the tracking period; the turtle moved its center of activity northward approximately 1 km, from one seagrass meadow to another seagrass meadow. Another individual (PTT 163693) appeared to use multiple seagrass meadow foraging sites, but distinct shifts were not identified by the segmentation analysis. During the first year of the tracking period, PTT 163693 used two foraging sites, then switched to using a single foraging site during the second year, shifting away from the seagrass meadow at which it had been captured 7 times during the previous 10 y (Fig 4C and 4D). For all other individuals, spatial and temporal shifts in use areas were apparent in visual examination of the Fastloc-GPS positions but were not detected by the segmentation analysis.

#### Cool weather refugia (c)

Cool weather refugia were in deeper water and were visited rarely or not at all during warm months.

**Fig 12:**
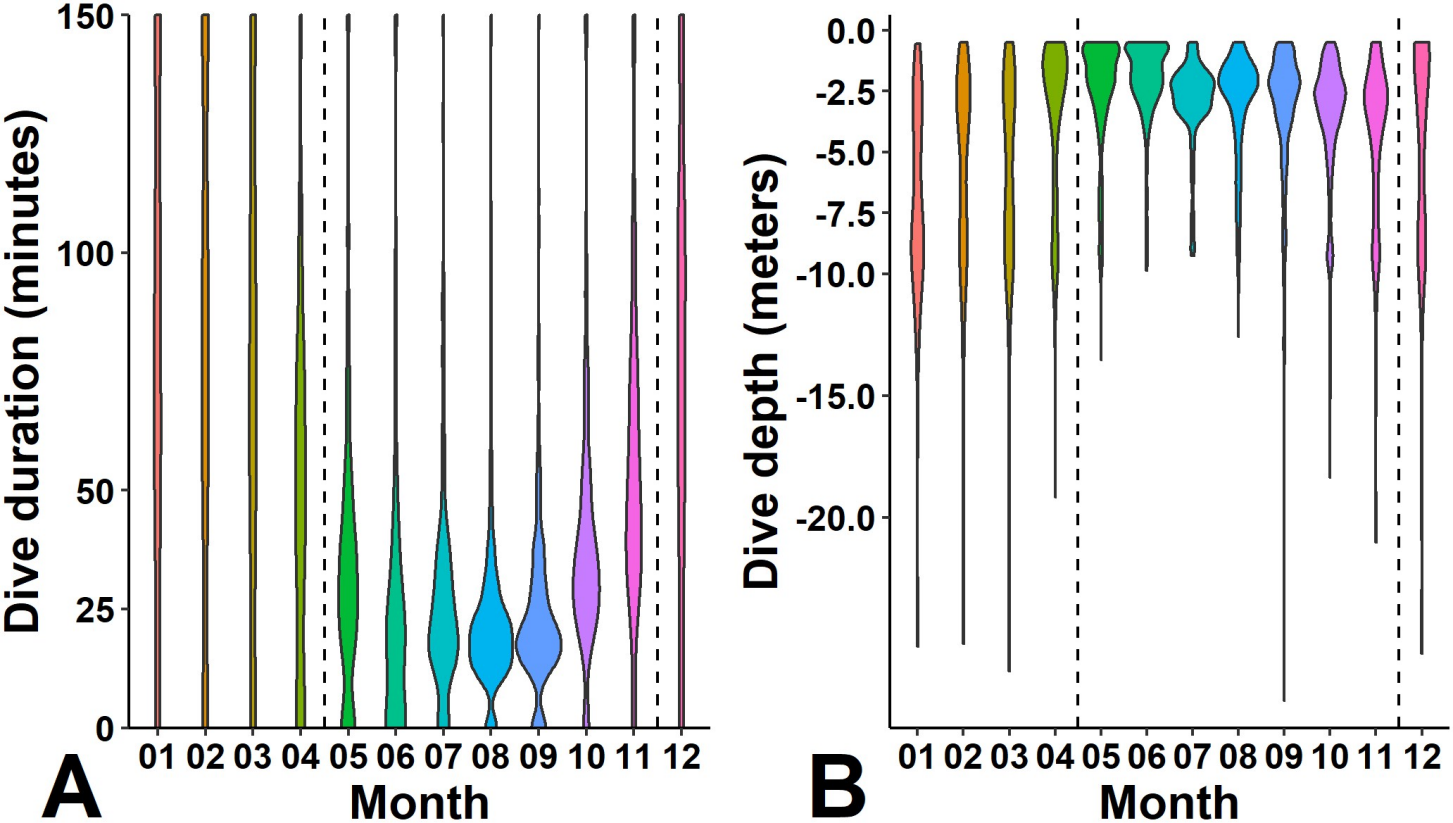
Dive duration (min, A) and depth (m, B) by month for 8 immature green turtles (*Chelonia mydas*) tracked in Bermuda with satellite transmitters equipped with dive sensors. These 8 turtles were tracked through all seasons. Dashed lines delineate cool and warm months. Depth data summarized from binned dive duration and time-at-depth data.

Water temperatures on the Bermuda Platform during 2009–2019 ranged seasonally from 15.7–31.2°C (mean 23.9°C). Mean monthly temperatures were highest from July–Sept (range: 28.2–29.2°C) and lowest from Jan–March (range: 18.6–18.9°C, Fig 2). We designated May–Nov as the warm season based on the tendency for water temperatures to be above 20°C during these months. We received diving behavior data during all seasons for 8 individuals and observed seasonal changes in diving behavior in all of them. During warm months, dives tended to be shorter in duration and shallower (Fig 12A and 12B). During cooler months, dive durations were more evenly distributed. Dive depths during the cooler months were bimodal as turtles used both shallow (<5 m) and deeper (5–10 m) depth ranges in roughly equal proportions. The average monthly dive durations ranged from 23.2 minutes (±19.3 SD) during Aug to 79.5 minutes (±51.1 SD) during Jan; dive depths ranged from 2.0 m (±1.6 SD) during June to 7.0 m (±4.2 SD) during Jan. Most dives were shallower than 5 m from April–Oct (Fig 12B). For four individuals with detailed dive profiles (Fig 3), changes in diving behavior occurred when the temperature crossed the 20°C mark, supporting our use of Dec to April as the “cool season.” Dive depths were negatively correlated with temperatures at the BEPB6-2695540 station, our proxy for near-shore, shallow-water temperatures, (mean r = −0.43, p < 0.05, Table 3) for all 8 individuals for which transmitters provided data during all seasons, indicating that dives tended to be significantly deeper when shallow-water temperatures were lower (Fig 12B).

All turtles tracked during cooler months increased their use of non-seagrass habitats during those periods. The percentage of time that turtles spent off seagrass meadows during daylight hours increased to 47.2% during cool months (compared to 20.4% during warmer months). Turtles that tended to spend nighttime hours on seagrass meadows increased their nighttime use of non-seagrass habitats (37.1% of time) during cooler months (compared to 15.8% of time during warmer months). Six individuals used distinct areas during cooler months which we referred to as cool weather refugia (c). Cool weather refugia were 0.29 km2 in average size and occurred at an average depth of 10.4 m. These sites were an average of 3.04 km from foraging sites (±2.41 SD, range: 0.99–7.67 km).

Distance moved from the mean center of the 25% kernel UD was negatively correlated with near-shore, shallow-water temperatures for 12 individuals (mean r = −0.29, p < 0.05, Table 3), indicating that movements away from the center of the primary foraging area were greater at lower temperatures. Two turtles exhibited significant positive correlation estimates (PTTs 163693 and 172209), indicating that departures from the primary foraging area were sometimes made during periods of very warm water temperatures. Those two individuals regularly used warm weather resting areas (r) and cool weather refugia (c) that were equidistant from their foraging areas (e.g., Fig 4).

We lacked in situ habitat characterizations of cool weather refugia. However, a review of satellite imagery and Fastloc-GPS positions collected during cool periods suggested turtles were principally using rocky outcroppings similar to those used for resting habitats during warmer periods but were at greater average depth. Green turtles that were tracked during cool months continued to visit seagrass meadow foraging areas and resting areas that they used during warm months. Their use of cool season refugia tended to be brief (days to weeks) and coincided with declines in water temperature. This suggests green turtles may forage nearly year-round in Bermuda, whenever temperatures on seagrass meadows are suitable. One turtle (PTT 163693) was tracked during two winters. This individual used the same cool weather refugium during both winters (Fig 4). This finding suggests green turtles may exhibit fidelity to resting and cool weather refugia over multiple years, similar to the fidelity they exhibit to seagrass meadow foraging habitat as has been observed in this and other studies.

### Long-term residency and detectability

Net capture data (red triangles in Figs 4 and 6–9), along with satellite transmissions, suggested residency of tracked green turtles on the Bermuda Platform for up to 181 months (Table 4). For 11 of 16 turtles that met the requirements for assessing detectability (see methods), all net captures (1–7) occurred within the residence area revealed by satellite telemetry. These turtles were captured one or more times in the presumed foraging area (f/r) in the years prior to and, in two cases, following transmitter deployment, and telemetry data indicated fidelity to a limited area of the Platform. Assuming fidelity to this site over an extended period, frequency of detection with the entrapment net varied from 0.063 to 0.667 (mean = 0.310 ±0.204 SD).

**Table 4:** Residency and detectability of satellite tracked green turtles (*Chelonia mydas*) on the Bermuda Platform. Total known residency time on the Platform is given and assumed to be continuous residency in one sampling area. Detectability at that site is given if residency was 1 yr or longer. Transmitter deployment details given in Table 1.

PTT	First capture date	Most recent Bermuda observation (net capture^n^, telemetry^t^)	Total Bermuda residency (months)	Bermuda capture location	Average distance between Bermuda captures (m)	Assumed residency at a single location	Net sets during residency period	Net captures during residency period	Rate of detection (%)
108507	8/4/2003	7/27/2012^t^	108	Blue Hole	106.9	yes	8	4	50.0
108508	8/12/2004	6/21/2012^t^	93	Vixen	323.1	yes	16	3	18.8
120325	7/30/2012	8/11/2014^n^	144	Tudor Hill & Wreck Hill	390	yes^a^	26	4	15.4
120326	8/13/2010	9/20/2012^t^	109	Annie’s Bay		yes	11	4	36.4
132092	8/7/2002	11/11/2013^t^	135	Bailey’s Bay	100.1	yes	31	6	19.4
132093	12/1/2003	8/15/2014^n^	140	Well Bay & Long Bay	190.4	yes^a^	10	2	20.0
140712	8/8/2014	9/25/2016^t^	25	Somerset Long Bay	one capture	yes	5	1	20.0
140713	8/8/2001	12/28/2015^t^	173	Tudor Hill & Wreck Hill	794.1	yes^a^	32	2	6.3
151800	8/14/2015	10/11/2015^t^	2	Fort St. Catherine	one capture	< 1 yr			
151801	8/17/2015	12/12/2015^t^	4	Wreck Hill	one capture	< 1 yr			
163691	8/14/2013	12/29/2016^t^	40	Somerset Long Bay	26.5	< 1 yr	8	2	25.0
163692	8/19/2015	7/10/2017^t^	23	Somerset Long Bay	83.7	yes	3	2	66.7
163693	7/28/2006	4/27/2018^t^	141	Long Bay	58.7	yes	11	7	63.6
172208	8/18/2017	7/11/2018^t^	11	Somerset Long Bay	one capture	< 1 yr			
172209	4/27/2004	6/5/2019^t^	181	Vixen & King Charles Hole	1,130.5	no			
174108	8/14/2013	6/5/2019^t^	70	Somerset Long Bay & Methelin Bay	584.2	no		4	
						**Rate of detection avg. ±SD = 31.0 ±20.4**			

^a^These turtles used both listed Bermuda capture locations during the tracking period.

### Departure from the Bermuda Platform

**Fig 13:**
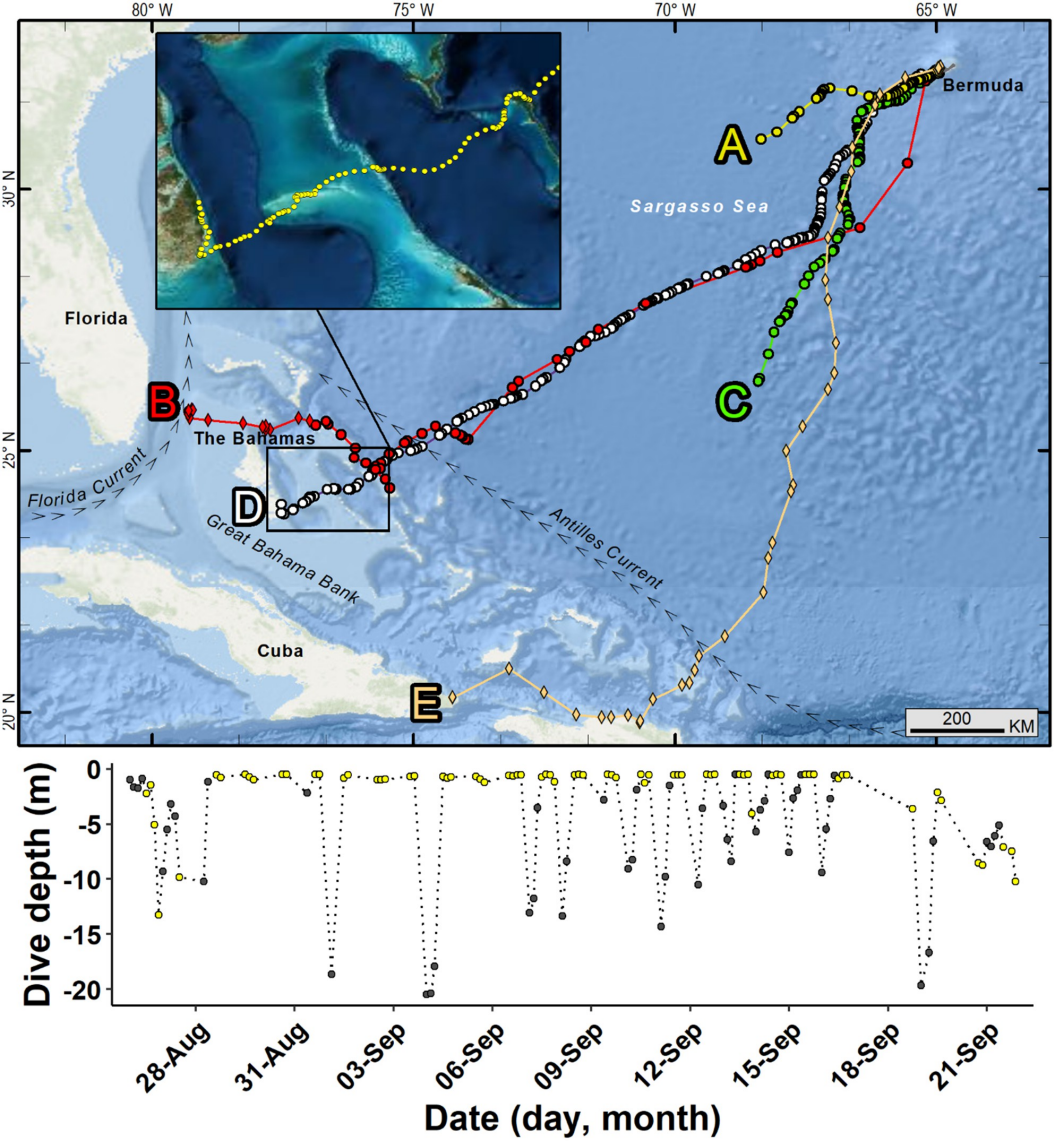
Developmental migrations made by five green turtles (*Chelonia mydas)* from Bermuda. Circles indicate Fastloc-GPS positions and diamonds Argos positions. A. (A) PTT 140712, (B) PTT 140713, (C) PTT 151801, (D) PTT 163691, (E) PTT 11674. Argos positions were used to display the end of the migration path of PTT 140713, during a period when no Fastloc-GPS positions were received. See Table 5 for details about tracks and individual turtles. Detail of (D), the final portion of the migration of PTT 163691 in the Bahamas during Sept 20–29, 2016 (inset). Lower panel shows daytime (yellow) and nighttime (black) dive depths recorded during the developmental migration of (D) PTT 163691. Republished from Esri, DigitalGlobe, GeoEye, Earthstar Geographics, CNES/Airbus DS, USDA, USGS, AeroGRID, IGN, and the GIS User Community under a CC BY license, with permission from Esri, original copyright 2022.

Four turtles departed the Bermuda Platform with an active transmitter (Table 5, Fig 13). Segmentation analysis revealed distinct pre-migratory behavior in all four turtles (S2 Fig), as well as departure dates for these individuals (Table 5). Three of the four made forays outside of their normal activity area during the days immediately preceding their departure (S2 Fig); pre-migratory movements involved 4–15 days of travel within areas that were larger than and distinct from their total residence areas. Data for one individual were intermittent during the months leading up to its departure which restricted our ability to characterize any distinct behavior during this period. Within this pre-migratory period, two individuals exhibited looping travel during which they departed and returned to their residence areas prior to beginning their developmental migration (S2A and S2D Fig). One individual traveled approximately 5 km west of its residence area, exhibited bi-directional movements for three days, then traveled southward, prior to beginning directed migratory travel (S2B Fig). All four individuals departed the Bermuda Platform during nighttime hours. The mean carapace length of turtles that departed was 66.1 cm SCLmin (±1.5 SD, range: 63.9–67.1). Turtles departed during June (1) [PTT 140712], Aug (2) [PTTs 140713 and 163691] and Sept (1) [PTT 151801]. Two turtles tracked in 2014 departed Bermuda during the following summer. Two others began their developmental migrations during the year in which the transmitter was deployed. These four departures are summarized below along with a developmental migration made by PTT 11674, an immature *C*. *mydas* satellite-tracked (Telonics ST-6) by the BTP during Aug 1998 [11]. This individual began its developmental migration 18 days following transmitter deployment.

**Table 5:** Developmental migration summary for five immature green turtles (*Chelonia mydas*) that departed from the Bermuda Platform. Path bearing, straightness, travel rate, and surface current velocity were calculated for locations in pelagic waters (depth > 200 m).

Track (Fig 11)	PTT	Deployment location (date)	Departure date	End of migration	Final location	Sex	SCLmin (cm)	Pre-departure foray	Path distance (km)	Path bearing (°)	Path straight-ness	Mean travel rate (km hr^-1^)	Surface current velocity (m s^-1^)	Mean dive depth (m, D = day, N = night)	N Fastloc-GPS locations
A	140712	Somerset Long Bay (8-Aug-14)	2-Jun-15	9-Jun-15	open ocean	U	66.8	Y	386	241.8 ±0.46	0.93 ±0.24	2.53 ±0.79	0.58 ±0.38		41
B	140713	Wreck Hill (11-Aug-14)	31-Aug-15	27-Oct-15	Bimini, Bahamas	M	63.9	N	2,025	254.2 ±0.59	0.96 ±0.08	2.20 ±0.69	1.35 ±0.73		40
C	151801	Wreck Hill (17-Aug-15)	8-Sep-15	24-Sep-15	open ocean	U	66.6	Y	800	210.1 ±0.60	0.90 ±0.28	2.25 ±0.70	0.79 ±0.33	13.2 ±4.9 (D), 13.8 ±8.0 (N)	76
D	163691	Somerset Long Bay (10-Aug-16)	27-Aug-16	29-Sep-16	Andros, Bahamas	F	67.1	Y	1,653	232.5 ±0.60	0.92 ±0.24	2.07 ±0.71	0.83 ±0.33	1.9 ±2.7 (D), 6.5 ±5.4 (N)	390
E	11674	Outside Daniels Head (8-Aug-98)	23-Aug-98	29-Sep-98	Baracoa, Cuba	F	78.6	U	2,043	218.0 ±0.85	0.92 ±0.23	2.28 ±0.98	0.83 ±0.36		34

All five individuals traveled in a southwestward direction upon departing the Bermuda Platform, path bearings ranged from 210.1°–254.2° (Table 5). Migrants traveled at an average rate of 2.27 km hr-1 (±0.17 SD). The turtles’ migratory paths were direct, path straightness indices ranged from 0.90–0.97 (mean = 0.93 ±0.02). Migrants did not appear to encounter strong surface currents during their transits, mean sea surface circulation velocity was 0.88 m s-1 (±0.29 SD, range: 0.58–1.35). Two of the migrants’ transmitters had dive depth sensors. One individual (PTT 163691) exhibited average dive depths that were shallow during daytime and deep during nighttime hours (day: 1.9 ±2.7 SD m; night: 6.5 ±5.4 SD m). The second (PTT 151801) exhibited deeper average dive depths that were similar during day and night (day: 13.2 ±4.9 SD m; night: 13.8 ±8.0 m). Migrating turtles’ daytime travel rates were significantly higher than nighttime travel rates (day: 2.4 ±0.7 SD km hr-1; night: 2.0 ±0.6 SD km hr-1; p < 0.05).

Two individuals are known to have completed a transit across the West Atlantic to the Bahamas (Table 5, Fig 13). Two others stopped transmitting at sea approximately 300 (PTT 140172) and 700 (PTT 151801) km SW of Bermuda. We found no evidence of transmitter battery failure [32] or mortality, as evidenced by cessation of movement and prolonged surface time [31]. Saltwater switch malfunction may have occurred in 7 of the transmitters used in the study. The previously tracked turtle, PTT 11674, also abruptly stopped providing transmissions because of capture by a fisher off the NE coast of Cuba while exhibiting directed travel.

One individual (PTT 163691) traveled to Andros Island, Bahamas, when transmissions abruptly stopped on Sept 29, 2016, between Deep Creek and the Bluff settlements. The tag began transmitting again on Nov 27, 2016; the new data suggested the tag was stationary and on land. We contacted colleagues in the region and arranged for the transmitter to be located and returned. The dive data recovered from the transmitter confirmed that it was below the surface of the water and stationary at a depth of 2–3 m from Sep 29–Nov 7. The transmitter may have detached from the turtle because of epoxy failure, or the turtle may have become entangled and held underwater. We did not learn the details surrounding the transmitter’s recovery.

The turtle that completed a transit to Bimini, Bahamas, established a residence area from which we received positional data for 57 days (Oct 27–Dec 23, 2015). The size of the total residence area falls within the range of variation seen for the 16 residence areas observed in Bermuda (Table 1, S3 Fig). It is unknown whether the turtle resumed travel after these data were collected or remained at this site for an extended period. The new residence area in Bimini was just a few km west of the proposed North Bimini Marine Protected Area (see Fuentes et al [40]).

## Discussion

We have assembled fine-scale habitat use information that complements the findings of long-term BTP research [11, 19] and advise conservation management for green turtles in Bermuda and similar benthic developmental sites worldwide. We have further confirmed fidelity to foraging and resting areas and identified several distinct behaviors of green turtles in Bermuda, including pre-developmental migration forays, fidelity to cool weather refugia, and changes in primary foraging grounds. The use of a large number of high-quality Fastloc-GPS positions (≥6 satellites, <30 residual error) for each tracked turtle provides a high level of certainty about movements and behavior. This allowed us to characterize habitat use beyond the level of home range to “distinct use areas” at the “patch use” level [41] in which fine-scale habitat use is revealed by multiple concentrations of points separated by 100s of meters or less. Dujon et al. [42] conducted trials with Fastloc-GPS transmitters in fixed locations and estimated that, when positions were derived from 6 satellites or more, 50% of locations are within 18 m of the true position and 95% are within 70 m. Our results include some general patterns shared among tracked turtles, but fine scale results also reveal important individual variation as has been found previously in green turtles and other sea turtle species [43]. Sea turtle satellite telemetry studies have focused largely on reproductive females of multiple species that were telemetered on nesting beaches. Males and earlier life stages have been underrepresented [44, 45]; our tracking study helps to fill this gap.

Comparing results of animal tracking studies is challenging due to technical and biological factors. Previous tracking studies of *C*. *mydas* have relied on a range of data acquisition systems (radio, acoustic, and satellite) and used a variety of estimators and descriptors of “home range.” Many studies describe a core use area, often derived from a 50% UD, and identify the full home range based on a 90 or 95% UD, or by using MCP. For satellite telemetry, there is the additional complication that studies have used locations estimated by the Argos Doppler shift method, Fastloc-GPS locations, or a combination of the two. Previous studies [41, 42, 46] have documented the advantages of GPS (especially Fastloc-GPS) for revealing fine-scale habitat use at foraging grounds. For green turtles in developmental habitat, a recent study in the Indian Ocean using only Fastloc-GPS [37] provides strong evidence for the utility of this technology for studies of this life history stage. In contrast, studies that have combined Argos with Fastloc-GPS positions positions have produced results that are useful at broad scales, *e*.*g*., Wildermann et al. [47], Siegwalt et al. [30], Metz et al. [48], Doherty et al. [49], but lack the precision and quantity of location data to allow comparisons at the level of distinct use areas, as in the present study. In a comparison of telemetry methods used with marine turtles, Thomson et al. [41] (Table 1) showed that both passive and active acoustic tracking have similar location accuracy to data from Fastloc-GPS, i.e., tens to hundreds of meters. Thus, along with Fastloc-GPS studies, results from acoustic tracking of juvenile green turtles in benthic developmental habitats in which locations were taken either by mobile observers [50, 51] or fixed arrays [52, 53] appear to provide more useful comparisons to our study than results based on Argos or combined Argos and GPS location data.

### Home range

Because all telemetered turtles exhibited behavior during the initial 24 h of deployment that did not appear representative of their typical movement patterns, the first 24 h of data were not used in home range estimations. The effects of instrumentation on study animal behavior may be particularly important when detailed habitat use patterns are a focus of study. This result may also be critical to understanding the use of animal-borne cameras to document turtle behavior. If the first 24 h of activity after capture is atypical, then any data collected during that period should be interpreted with extreme caution.

In this study, total residence area (MCP) included all presumed foraging, resting (day or night), and seasonally used areas. Its size was likely driven by biotic and abiotic habitat features such as forage availability, access to suitable depth and bottom structure for resting, and seasonal water temperature fluctuations, as has been described in previous studies, *e*.*g*., Seminoff et al. [54]. We observed no correlation between 90% UD (home range) and turtle size. Other studies have reported a positive relationship between turtle size and home range size [43, 53]. The absence of correlation in our study may be due to the narrow range of sizes of tracked turtles (48.4–69.4 cm SCL), rather than a sampling of the full range of neritic juveniles (25–80 cm) that occur in Bermuda. As reported by Chambault et al. [37] we found limited influence of deployment duration or number of locations on kernel estimation. Our home range estimate did increase in recent years (Table 1) which may indicate that turtles were broadening their foraging areas because of declining seagrass [19, 55–58].

The average size of home ranges (90% UD) in the present study (2.11 km^2^) compares well with estimates for green turtles made using mobile acoustic (or acoustic plus radio) tracking by Mendonça (3.49 km^2^, 100% MCP) [50]; Makowski et al. (2.09 km^2^, 95% UD) [51] and Renaud et al. (2.3–31.2 km^2^, 95% MCP) [59]. Our home range estimates were smaller than those for green turtles from the sounds of North Carolina (overall UD 39.7 km^2^ from acoustic telemetry and 84.6 km^2^ from Argos data) [60], saltwater creeks in the Florida Everglades (154.4 km^2^ 95% Kernel Density Estimate from Argos data), and seagrass meadows in the northeast Gulf of Mexico (24.3 km^2^ 95% UD from combined Argos and Fastloc-GPS data). The differences between our findings and those from other studies are likely due to differences in both habitat and the accuracy of the telemetry method employed.

### Distinct use areas

Although useful for delineating the overall area used, estimates of total residence area and home range size using traditional methods may fail to reveal distinct use areas that are biologically important. Distinct use areas in our study (Table 3) were smaller than high-use area estimates for green turtles in the Indian Ocean provided by a traditional home range estimator. Diurnal 50% UD home ranges estimated in two recent studies were 0.18 km2 for “juveniles” [37] and 3.6 km2 for adults [46]. Both were much larger than distinct use area size estimates in the present study (Table 3). Although the difference may be due to variation in turtle size or habitat characteristics, it is more likely methodological because we quantified diurnal foraging and diurnal resting areas separately. We were unaware of comparable studies in which separate distinct use areas within a juvenile green turtle home range were visually identified from discrete clusters of Fastloc-GPS locations.

Fidelity to seagrass meadow foraging sites by immature *C*. *mydas* is well documented (*e*.*g*., Ogden et al. [61], Hart and Fujisaki [62], Meylan et al. [11], Griffin et al. [63], Chambault et al. [37]). In 13 of 16 turtles, we observed evidence of foraging at a single site, visited daily, that was also the seagrass meadow on which the individual was captured. Previous publications on this aggregation reported that since 1979, 90% of recaptures of marked individuals were made on the same seagrass meadow as the original capture [11, 19]. In-water net capture data for tracked turtles support the telemetry results. One tracked turtle had been captured on the same seagrass meadow 7 times in 10 years (Fig 4D); another had been captured 6 times over 11 years (Fig 7B). Although individuals in our study exhibited daily fidelity to specific foraging sites, this pattern may be changing. More recently tracked turtles had larger home ranges and an individual tracked from 2016–2018 changed foraging areas (Fig 4). We also observed individual variation in daily time use patterns of seagrass meadows (Fig 10). This variation may reduce intraspecific competition for foraging or resting resources.

Previous studies have characterized daytime and nighttime resting sites away from seagrass meadows used by green turtles as being located at patch reefs near the seagrass meadow [46, 51, 61, 64].

In our study, both daytime and nighttime resting areas away from the foraging area occurred in deeper water and were typically characterized by reef or rocky habitats at an average depth of 8 and 7.9 m (daytime and nighttime r, respectively). Turtles appeared to rest near the bottom based on dive data collected when turtles were at resting sites. Makowski et al. [65] similarly found that green turtles exhibited deeper dives during nighttime resting periods. Our results support the hypotheses that green turtles may choose resting sites based on depth [66] and structure [67]. Previous observations of green turtles making mid-day departures from foraging areas and traveling to nearby resting sites [50, 61] support our finding that some turtles rest away from foraging areas. All tracks included nighttime points at the foraging site indicating that some nighttime resting (and possibly nighttime foraging) occurs at the foraging site (Fig 10).

Similar to the pattern observed for “juvenile” green turtles in the Indian Ocean [37], we found the total area used by turtles during resting periods was smaller than the foraging area. In one exception (PTT 132092) the turtle appeared to use multiple resting areas adjacent to the seagrass meadow on which it foraged (Fig 7). No turtles used the same daytime and nighttime resting areas away from the seagrass meadow, which suggests that turtles may have differing resting habitat preferences during the two periods (see also Chambault et al. [37]). This difference could be due to turtles selecting daytime sites based on proximity to a foraging area versus reduced predation risk at night. Additionally, anthropogenic disturbance may be a factor in resting habitat selection [37] and could have significant impact in Bermuda where nearly all recreational boating occurs during daylight hours. Forage availability, habitat structure, and anthropogenic disturbance may all factor into the selection and size of distinct use areas for foraging, daytime resting, and nighttime resting by juvenile green turtles in Bermuda.

Meylan et al. [11] showed that green turtles in Bermuda are not “itinerant” but rather reside on the Bermuda Platform year-round, for many years. Given the high latitude of the site, we expected to observe changes in behavior during periods of cool water temperature [68]. For 7 cases, where seasonal refugia (c areas) could be spatially delimited, the refugia were larger than f or r areas, deeper (10 m mean depth), and farther from seagrass meadow foraging sites than r areas (3.04 km mean distance vs <1 km). In 4 of 10 tracks that included cool weather months, significant seasonal movements were detected by the Segmentation Analysis, in the remaining six cases they were not. The Segmentation Analysis likely failed to detect distinct c areas for all turtles tracked during cooler months for several reasons, including limited Fastloc-GPS positions, brief usage of c areas during some winters, or partial overlap with other use areas.

### Overwintering

Sea turtles appear to have four different overwintering options: dormancy or brumation, migration to lower latitudes, return to pelagic/oceanic state [69] or “optional dormancy” [70]. Evidence for true dormancy is limited [70, 71]. Ultsch [71] considers hibernation in sea turtles to be controversial and suggested that brumation might be a better term. Meylan et al. [11] reported captures of green turtles on seagrass meadows in Bermuda during all months. The turtles followed through winter months in the present study were regularly located on seagrass meadows during cool months (Figs 4, 6, 8 and 9). Thus, long-term brumation does not seem to be occurring. Southerly movement along a U.S. coastline is documented for green turtles in North Carolina [69] and Texas [48], but in the case of Bermuda, there is no other suitable neritic habitat within 1,000 km. We have yet to observe any evidence that the smallest size classes (or any size classes) are returning to surface-pelagic environments as has been documented in North Carolina, USA [69]. The Bermuda Platform is hundreds of km east of the Gulf Stream Current so there is no reliable nearby source of warm water from lower latitudes. Thus, *optional dormancy* [70] seems to be the best description for the overwintering strategy of green turtles in Bermuda. Tracked turtles tended to move away from shallow, nearshore waters when temperatures dropped below about 20°C and occupied deeper sites that averaged more than 10 m in depth (Table 2). While in cool weather refugia, water temperatures reported by transmitters were warmer and less variable than those in shallower, nearshore waters. Turtles returned to making shallower dives when temperatures were only a few degrees higher (Fig 3).

Sea turtles are known to exhibit behavioral responses to water temperature fluctuations especially at high latitudes where seasonal temperature fluctuations are more dynamic. For example, Schofield et al. [72] found that nesting loggerheads at high latitudes exploit patches of warm water early in the nesting season. Additionally, previous studies have demonstrated reduced metabolic activity and longer dive durations during winter months (e.g., [73]). Summaries of diving behavior in our study (Fig 12) showed increases in dive depth and duration during winter months. There was a marked decrease in the time spent at shallow depths during winter months, but turtles also continued to make regular visits to seagrass meadows throughout the winter, which suggests the possibility that some feeding may still occur in cool months. Manuel et al. [58] noted that the depth at which *T*. *testudinum* was most common was at the shallowest sites they surveyed (< 2 m). Therefore, if turtles are foraging during winter months, they are likely to return to shallow waters to do so. If the purpose of seasonally used sites is primarily for access to the thermal stability of deeper water during cold snaps, the distance moved during these periods may be a function of the length and severity of drops in water temperature and the proximity of sufficiently deep water. A similar pattern of movement to deeper water during winter was noted for green turtles in developmental habitat in St. Joe Bay, Florida [8]. Most individuals that were observed using cool weather refugia appeared to find refugia at multiple sites that had similar characteristics (depth, distance from seagrass meadow). One individual that was tracked in Bermuda during two winters returned to previously used c sites during the second winter indicating fidelity to overwintering sites as reported by Broderick et al. [5].

### Developmental migrations

Because green turtles in Bermuda are long-term but not permanent residents during their juvenile developmental life stage, we anticipated that some telemetered individuals would undertake developmental migrations away from Bermuda as was previously observed [11]. All five observed developmental migrations (Table 5) began during summer months, when ocean temperatures are warm. This differs from seasonal coastal migrations by juveniles moving away from their foraging sites, which have been reported to be triggered by marked decline in water temperature and occurred during winter months [48, 69]. Before departure, three individuals exhibited pre-departure behavior that was distinct from the previously described patterns of activity within their home range. Other studies have reported brief departures from foraging areas by larger juvenile green turtles [52] that might also include these pre-departure forays.

Mean straightness of travel for the 5 turtles that made a developmental migration was 0.926 ±0.022 SD (Table 5). This is much higher than has been observed for adult green turtles (0.77 ±0.13 SD) or adult hawksbills (0.55 ±0.23 SD) making reproductive migrations in the Indian Ocean [74]. Turtles departing Bermuda for foraging grounds in the Bahamas or Caribbean may not encounter strong western boundary currents that could affect path straightness until they reach the Antilles Current as they approach the Eastern Caribbean islands or the Bahamas. Reductions in path straightness of sea turtle migrations caused by their crossing strong currents has been previously documented [75]. The absence of strong currents may also explain higher travel rates compared to previous studies [76]. Because we did not observe the completion of these developmental migrations, with the exception of (PTT 140713, S3B Fig), we expect that overall path straightness would have declined as migrating turtles encountered the Antilles Current, the Gulf Stream System, and land masses while traveling to their next foraging area.

It seems unlikely that green turtles emigrating from Bermuda feed during their developmental migrations. Smaller green turtles (26.7–35 cm) that depart the North Carolina shoreline for the Gulf Stream during winter appear to have this option [69] but these larger individuals (63.9–78.6 cm) emigrating from Bermuda do not seem to return to surface pelagic feeding and do not appear to slow down to eat during their open ocean crossings. Instead, we have observed an occasional slowdown in the rate of travel when turtles cross possible benthic foraging habitat. PTT 163691 slowed considerably while crossing shallow banks near Cat Island and Scrub Cays in the Bahamas (Fig 13), and PTT 11674 slowed down while passing over the Silver Banks. PTT 140713 established new shallow-water foraging and resting areas once it reached suitable habitat in Bimini (S3B Fig).

Departure in a southwestwardly direction (210.1°–254.2°) by all 5 turtles (Fig 13, Table 5) is consistent with the hypothesis that marine turtles of several species migrate to sites closer to their natal beach as they approach maturity [11, 77]. Green turtles tagged in Bermuda as immatures have been found nesting years later on beaches in Costa Rica, Mexico, and Florida [78, 79]. The majority of international tag returns of green turtles tagged as immatures in Bermuda have come from Nicaragua, Cuba, and Venezuela [11], which are known adult foraging areas for *C*. *mydas* rookeries. Control region haplotypes (740 bp) are available for four of the five turtles shown in Fig 13. The turtle that took the most southerly course (track E) is the only Cm A3.1, the most common haplotype at Tortuguero, Costa Rica. Three others that took more westerly courses (tracks A, B and D), were Cm A1.1 (n = 2) and Cm A17.1, haplotypes known from Florida and Quintana Roo, Mexico rookeries but not from Tortuguero.

Our review of transmitted data revealed no evidence of transmitter battery expiration or biofouling. We conclude that the most frequent cause for the end of deployment was transmitter detachment due to epoxy failure. This finding suggests that we could have increased data collection and transmission schedules without exhausting transmitter batteries. We recommend biofouling mitigation measures and further investigation of attachment techniques that are more persistent while remaining safe for the telemetered animals.

### Detectability

Few studies of marine turtles utilize the entrapment net method employed by the BTP and, to our knowledge, estimates of detectability using this method are not available. The high degree of variation in detectability, from 0.063 to 0.667 (Table 4), is best explained by the size of the sampled seagrass meadow foraging areas relative to the area enclosed by the entrapment net (0.03 km^2^, Meylan et al. [19]). Higher detectability occurred in seagrass meadows of smaller size (Fig 4D) versus those with extended continuous habitat (Fig 6A) in which it was less likely to include the exact f area of a specific turtle. Satellite tracked turtles often departed foraging areas for daytime resting sites during mid-morning hours and returned to the foraging sites during the afternoon (Fig 10). For safety and logistical reasons, BTP capture efforts typically began at 8–10 AM and concluded by 2–4 PM which may have resulted in reduced captures of animals at their foraging site. Estimates of survivorship may assume animals that are not recaptured have emigrated or perished; however, a potentially large proportion of the cohort may have simply been unavailable for capture on any given day because of the timing of capture efforts relative to this bimodal pattern of behavior. This phenomenon has the potential to affect demographic estimates derived from CMR studies [78, 79]. It is also important to note that turtles tracked in the present study were larger (62.3 ±5.0 SD cm SCL min) than the average size of green turtles captured in Bermuda (annual average 38.6 to 43.3 cm during years of this study; [19]). It is possible that smaller juvenile turtles differ from larger, immatures in the time(s) of day they forage, or the amount of time spent foraging.

### Conservation considerations

Satellite telemetry studies involving protected species should be designed to address conservation needs [45]. In this study, turtles exhibited fidelity to multiple discrete use areas (f, r, c) illustrating the need to identify and protect all portions of the residence area (MCP) including transit corridors.

Results of this study are relevant to mounting concerns about the impact of seagrass decline on green turtle populations [55–58]. They support one of the main conclusions of a recent paper on changes in the green turtle aggregation in Bermuda over five decades that reduced site fidelity is likely to be a response to seagrass decline across the Bermuda Platform [19]. Both studies observed turtles changing foraging sites, and in this study, we documented significant increase in home range size (90% UD) during a recent period of documented seagrass decline [19]. Climate dynamics apparently have had negative impacts on Bermuda’s seagrass meadows; a recent cold weather event may have triggered initial seagrass loss [55]. In the present study green turtle foraging activity was highest during the warmer months of April to Nov (Fig 12). This suggests that documented ocean warming in the North Atlantic [80] is likely to result in increased foraging opportunities during winter months. If foraging can occur year-round at this and other more temperate foraging grounds, it is certain to put additional pressure on seagrass resources.

Vessel strikes and entanglement in monofilament fishing lines are the most frequently documented threats to sea turtles in Bermuda [81]. Vessel strikes can be a significant mortality factor for sea turtles anywhere that sea turtles and vessel traffic overlap [82]. The 90% UD of 10 turtles in our study intersected with a major boat navigation channel; 9 of these were along the western shoreline of Bermuda and one was in the Great Sound, near a cruise ship terminal (e.g., Figs 6, 8 and 9 and S3A Fig). All are areas of high vessel traffic. In multiple cases, turtles were recorded making twice-daily transits between seagrass meadows and deeper resting sites that involved crossing these heavily used channels. Turtles foraging at seagrass meadows along the western shore of Bermuda may be particularly vulnerable (Figs 6, 8 and 9 and S3A Fig). Boat moorings can damage seagrass habitat and are an additional threat related to vessel activity [83, 84]. Conservation measures designed to reduce vessel strike risk in green turtles would be most effective if they consider foraging, resting, and cool weather refugia, as well as travel corridors among those habitats.

Green turtles making developmental migrations from Bermuda encounter additional threats during their southward transits across the Sargasso Sea in the North Atlantic Ocean, *e*.*g*., pelagic longline fisheries, recreational and commercial vessel traffic, pollution and marine debris. These long-distance and international transits illustrate the need for broad, multinational marine conservation programs such as the Sargasso Sea Alliance [85, 86].

The ability to document sea turtle behavior at fine scale should greatly enhance the effectiveness of management strategies. This may be the most important advantage of Fastloc-GPS-enabled telemetry over Argos or acoustic tracking. The behaviors documented here are likely shared by juvenile green turtles elsewhere in the western Atlantic, particularly those using seagrass meadow foraging habitats at higher latitudes. The conservation implications of the present study should therefore be applicable elsewhere in the range of green turtles.

## Supporting information

S1 Fig Initial 7 days of movement data for two green turtles (*Chelonia mydas*) (A) 163691 and (B) 163692 that departed from their presumptive foraging area in Bermuda immediately after deployment and returned to their capture vicinity by day 7. Republished from Esri, DigitalGlobe, GeoEye, Earthstar Geographics, CNES/Airbus DS, USDA, USGS, AeroGRID, IGN, and the GIS User Community under a CC BY license, with permission from Esri, original copyright 2022.
(TIF)

S2 Fig Predeparture forays made by four green turtles (*Chelonia mydas*) that emigrated from Bermuda during their tracking period. Open diamond indicates deployment site, Fastloc-GPS locations (circles) are coded for day (yellow) or night (black), and travel direction is indicated. Tracks are shown up to the point where the turtle passed over the 200m isobath (black line). (A) PTT 140712, (B) PTT 140713, (C) PTT 151801, (D) PTT 163691. Remainder of each track is shown in Fig 13. Bathymetric contours provided by the Bermuda Department of Environment and Natural Resources; selected contours are labeled (depth m).
(TIF)

S3 Fig Fastloc-GPS locations (circles) and home range utilization distributions (25%—yellow, 50%—orange, and 90%—red polygons) of PTT 140713 on an immature green turtle (*Chelonia mydas*), (A) Wreck Hill, Bermuda, Aug 11, 2014–Oct 27, 2015 and (B) North Bimini, Bahamas, Oct 22–Dec 28 2015, after completing transit from Bermuda (Fig 13). In Bermuda, two distinct use areas were revealed: the primary foraging and resting area (f/r) used during all months and during both daytime and nighttime hours, and a resting area (r) used during daytime during all months prior to emigration. Locations are symbolized for daytime and nighttime. Republished from Esri, DigitalGlobe, GeoEye, Earthstar Geographics, CNES/Airbus DS, USDA, USGS, AeroGRID, IGN, and the GIS User Community under a CC BY license, with permission from Esri, original copyright 2022.
(TIF)
