# Supplementary figures and images for: Daily, seasonal, and long-distance movements inferred from Fastloc-GPS telemetry of immature green turtles (Chelonia mydas) at a high-latitude, mid-ocean developmental site
Source: PLoS One. 2023 Dec 15;18(12):e0292235. doi: 10.1371/journal.pone.0292235 (PMC10723664; doi:10.1371/journal.pone.0292235)

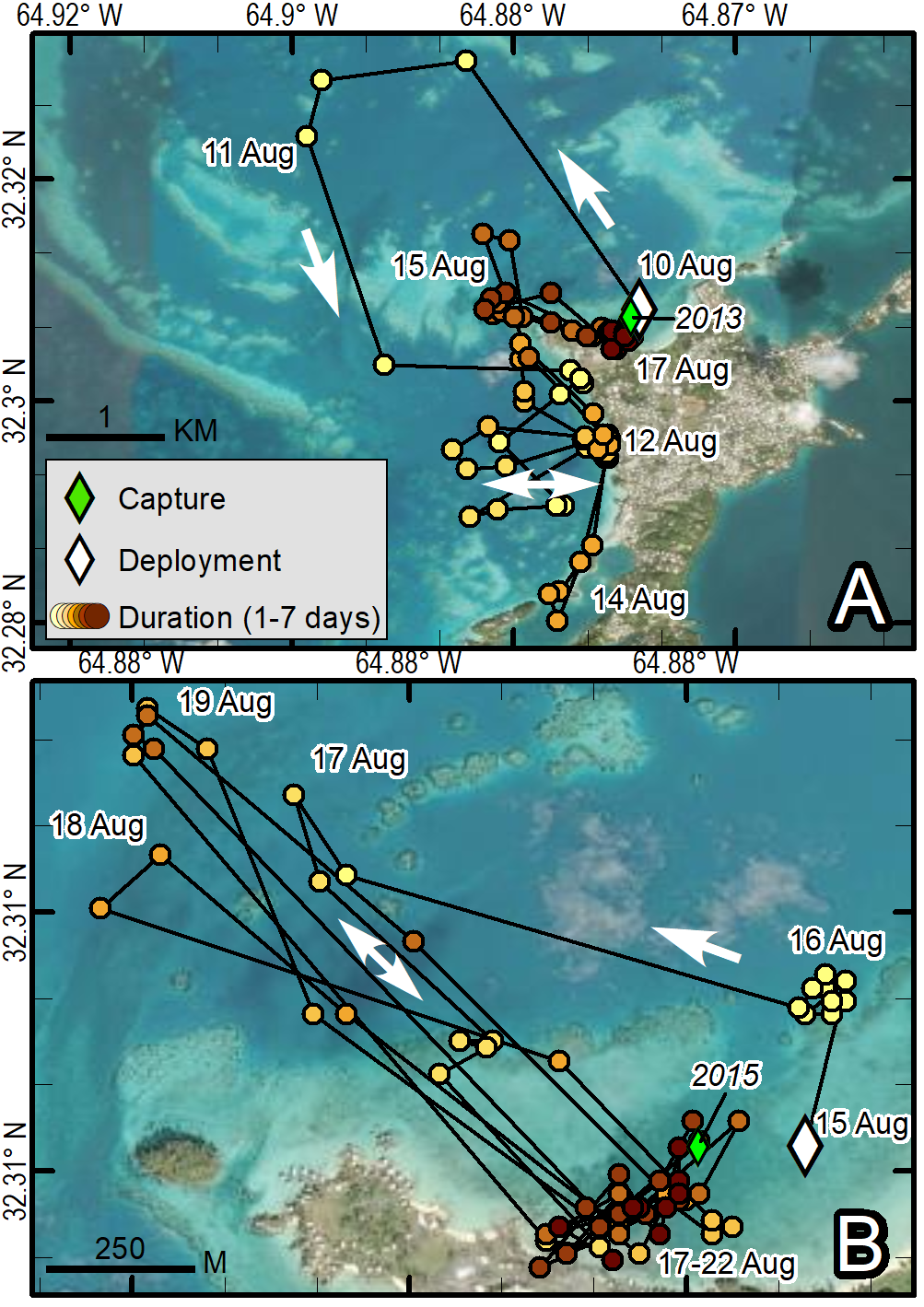

Supplement: S1 Fig — Initial 7 days of movement data for two green turtles (Chelonia mydas) (A) 163691 and (B) 163692 that departed from their presumptive foraging area in Bermuda immediately after deployment and returned to their capture vicinity by day 7. Republished from Esri, DigitalGlobe, GeoEye, Earthstar Geographics, CNES/Airbus DS, USDA, USGS, AeroGRID, IGN, and the GIS User Community under a CC BY license, with permission from Esri, original copyright 2022. (TIF) [file pone.0292235.s001.tif]

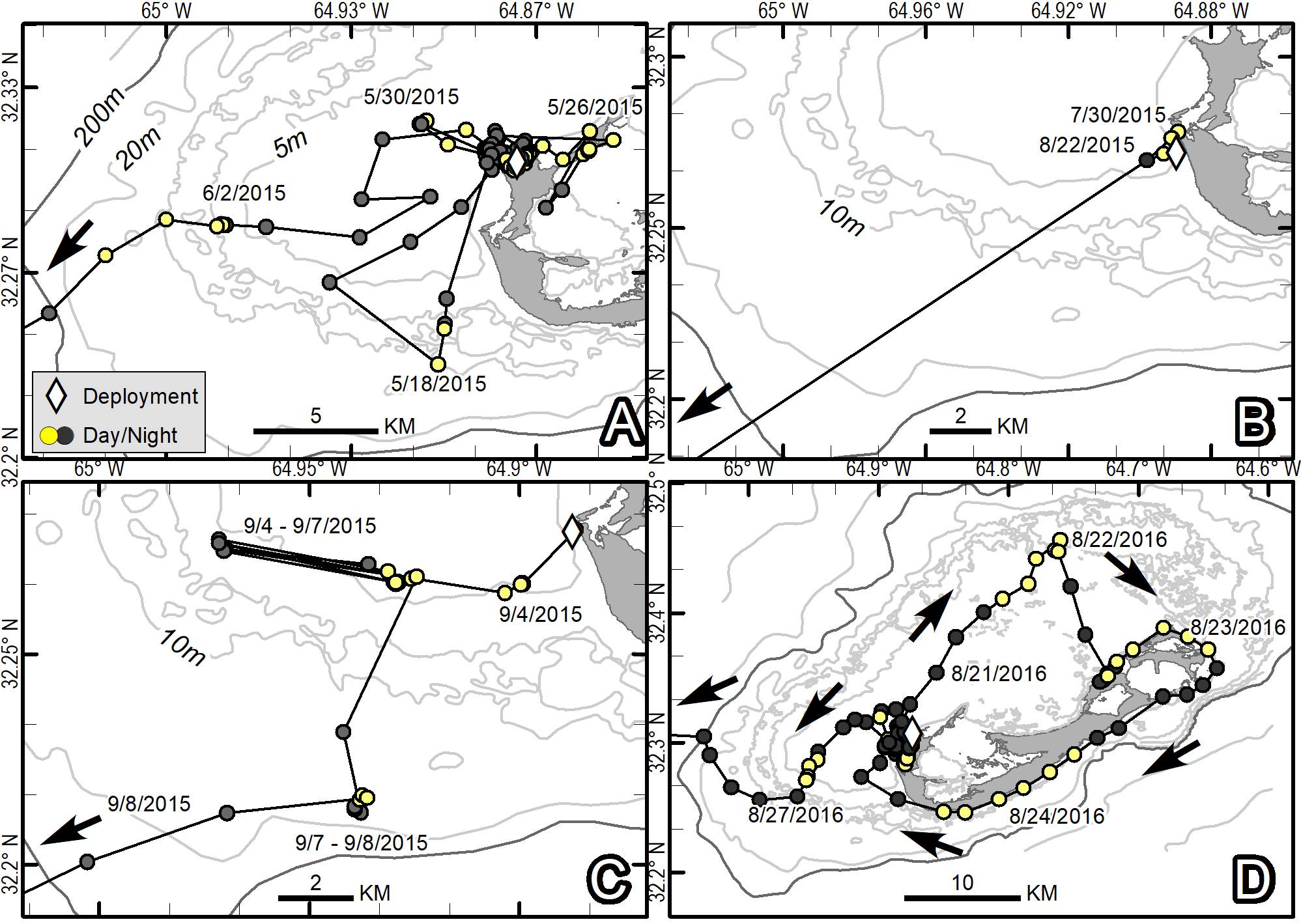

Supplement: S2 Fig — Open diamond indicates deployment site, Fastloc-GPS locations (circles) are coded for day (yellow) or night (black), and travel direction is indicated. Tracks are shown up to the point where the turtle passed over the 200m isobath (black line). (A) PTT 140712, (B) PTT 140713, (C) PTT 151801, (D) PTT 163691. Remainder of each track is shown in Fig 13. Bathymetric contours provided by the Bermuda Department of Environment and Natural Resources; selected contours are labeled (depth m). (TIF) [file pone.0292235.s002.tif]

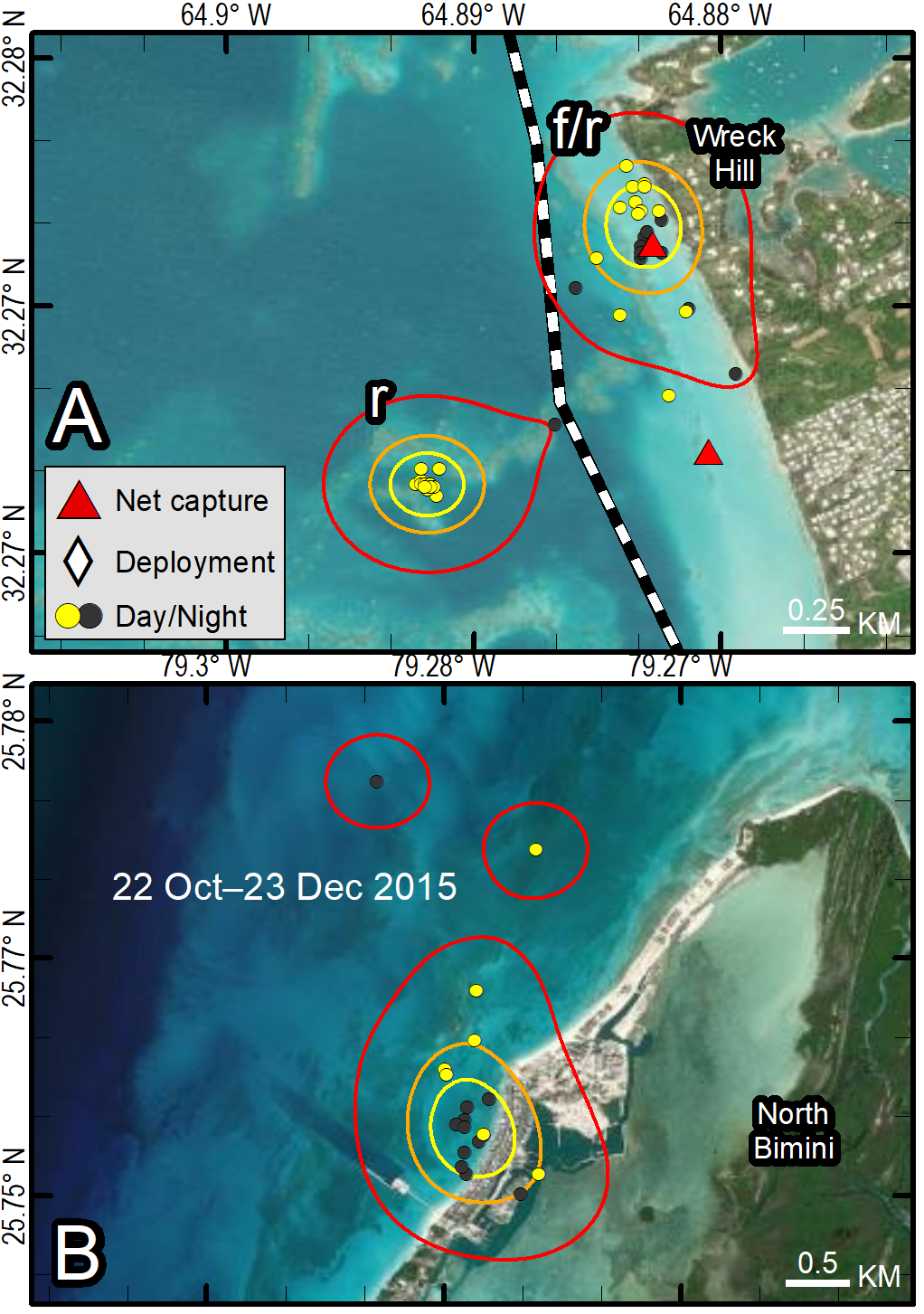

Supplement: S3 Fig — Fastloc-GPS locations (circles) and home range utilization distributions (25%—yellow, 50%—orange, and 90%—red polygons) of PTT 140713 on an immature green turtle (Chelonia mydas), (A) Wreck Hill, Bermuda, Aug 11, 2014–Oct 27, 2015 and (B) North Bimini, Bahamas, Oct 22–Dec 28 2015, after completing transit from Bermuda (Fig 13). In Bermuda, two distinct use areas were revealed: the primary foraging and resting area (f/r) used during all months and during both daytime and nighttime hours, and a resting area (r) used during daytime during all months prior to emigration. Locations are symbolized for daytime and nighttime. Republished from Esri, DigitalGlobe, GeoEye, Earthstar Geographics, CNES/Airbus DS, USDA, USGS, AeroGRID, IGN, and the GIS User Community under a CC BY license, with permission from Esri, original copyright 2022. (TIF) [file pone.0292235.s003.tif]
